# Early differences in neural adaptation to written words before reading instruction in dyslexia

**DOI:** 10.1016/j.dcn.2026.101815

**Published:** 2026-09-10

**Authors:** Anna Banaszkiewicz, Katarzyna Chyl-Tanaś, Hanna Górecka, Karolina Zielińska, Anna Redeł, Anna Szukało, Agnieszka Dębska, Katarzyna Jednoróg

**Affiliations:** a Laboratory of Language Neurobiology, Nencki Institute of Experimental Biology, Polish Academy of Sciences, Pasteur St. 3, Warsaw, 02-093, Poland; b The Institute of Educational Research, Górczewska St. 8, Warsaw, 01-180, Poland

**Keywords:** Dyslexia, Literacy acquisition, Neural adaptation, Repetition suppression, Neuroimaging

## Abstract

Reduced neural adaptation has been proposed as a neurophysiological characteristic of dyslexia, but it remains unclear whether such differences precede reading acquisition or emerge as a consequence of limited reading experience. Prior studies have focused on literate individuals, limiting developmental inferences. In this preregistered longitudinal fMRI study, children oversampled for dyslexia risk were scanned in kindergarten (prior to formal reading instruction) and again in third grade. Using the rapid adaptation paradigm, we examined neural adaptation to written words, false fonts, faces, houses and objects. Analyses focused on whole-brain effects and predefined regions of interest (ROI), including ventral occipito-temporal cortex (vOT). Dyslexic and control groups were case-control matched on age and reading skill at kindergarten. In ROI analyses, children who later developed dyslexia compared to controls showed reduced neural adaptation to written words in the left vOT already in kindergarten, despite matched pre-reading skills. This result was confirmed in individually-defined ROI analyses and was restricted to the anterior part of the vOT. The group difference remained stable across development. Across the full kindergarten sample (N = 59), adaptation to words in left vOT was modestly associated with prereading skills, including phonological awareness. No robust group differences were observed at the whole-brain level or for non-linguistic visual categories. These findings indicate small, region-specific differences in neural adaptation to print that are detectable prior to formal reading instruction. The absence of whole-brain effects and for non-linguistic visual categories suggests that neural adaptation differences in dyslexia are localized and domain-specific.

## Highlights

• Neural adaptation tested in control and dyslexic children in kindergarten and Grade 3
• Left (anterior) vOT differences in adaptation to words were observed
• Left vOT word adaptation correlated with reading and phonological skills
• No evidence for global neural adaptation deficits in dyslexia

## Graphical Abstract

**Figure:**
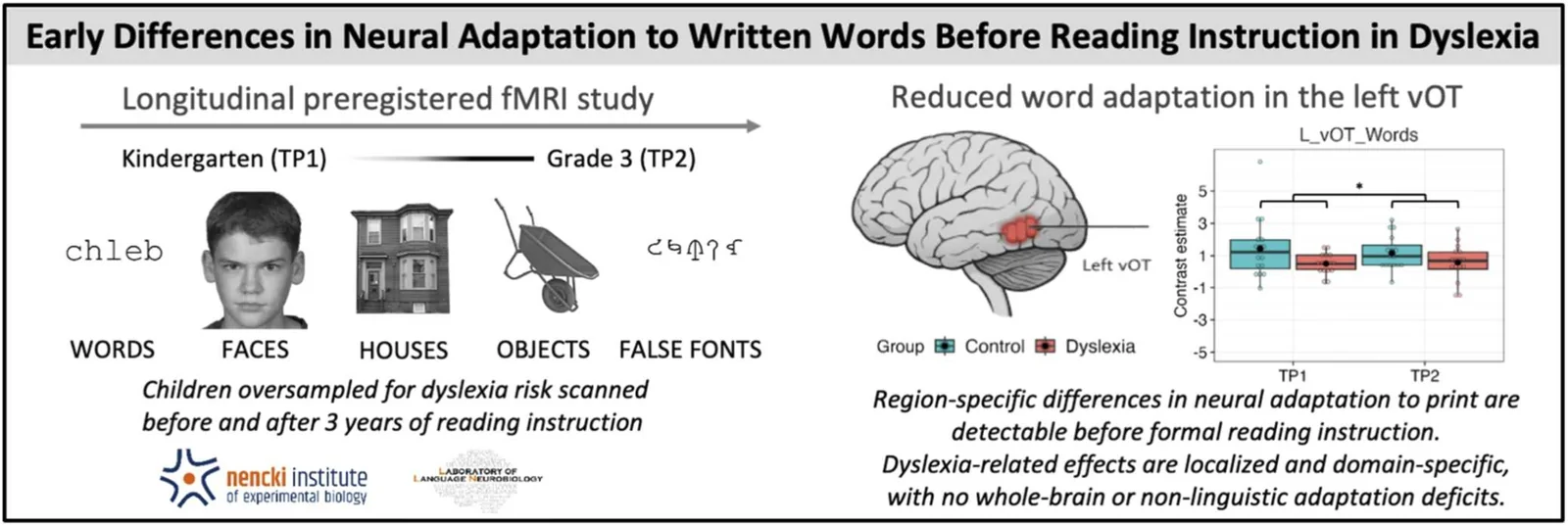

## 1 Introduction

A dysfunction in neural adaptation has been proposed as a potential neurophysiological marker of dyslexia contributing to impaired reading development (Perrachione et al., 2016). Neural adaptation refers to the reduction in neural responses following repeated exposure to the same stimulus, reflecting both the selectivity of brain regions for specific stimuli (Grill-Spector and Malach, 2001) and the plasticity involved in learning from repeated exposure (Berlot et al., 2021). In this context, adaptation deficits describe a reduced decrease in neural activity with stimulus repetition, indicating less efficient learning or tuning to repeated information. Using an fMRI rapid adaptation (fMRI-RA) paradigm, Perrachione and colleagues (2016) assessed neurophysiological adaptation to stimulus repetition in adults and children with dyslexia, compared to age-matched controls. In adults, they examined adaptation across spoken and written words as well as non-linguistic visual stimuli (faces and objects), while in children, only spoken words were tested. They reported diminished neural adaptation in individuals with dyslexia in stimulus-specific cortical areas: in adults across all stimulus types, and in children for speech. Better reading skills in adults and improved phonological awareness in children with dyslexia were linked to greater repetition-induced neural adaptation to speech in the left planum temporale. However, no such relationship was observed for written words in the ventral occipito-temporal areas. They concluded that reduced neural adaptation may reflect a domain-general processing deficit and posited it as a potential causal mechanism of dyslexia.

Subsequent studies have produced mixed evidence. For example, Gertsovski and Ahissar (2022) reported diminished auditory adaptation in adults with dyslexia to repeated tones in the auditory cortex, superior temporal and left parietal regions, consistent with the idea that difficulties in “anchoring” to stable stimulus patterns contribute to their reading difficulties (Ahissar, 2007, Ahissar et al., 2006). However, two recent studies, one using auditory syllables (Beach et al., 2022) and the other written words (Tan et al., 2022) found adaptation effects in adults with dyslexia that were quantitatively and qualitatively similar to those of typically developing readers, inconsistent with the hypothesis that the brain is less sensitive to repetition in dyslexia. In the latest fMRI rapid adaptation study, Wójcik et al. (2026) examined children aged 10–14 years with dyslexia and typically reading controls, finding evidence of reduced phonological adaptation in the left temporoparietal cortex in dyslexia, while orthographic adaptation in the left anterior ventral occipito-temporal cortex was comparable between groups.

All prior studies focused on already literate individuals, making it difficult to determine whether reduced neural adaptation is a precursor to dyslexia or a consequence of limited reading experience. To address this gap, we aimed to provide novel longitudinal data by following children oversampled for those at risk for dyslexia, from kindergarten to third grade, when a formal dyslexia diagnosis was conducted. Using an fMRI-RA paradigm, we examined adaptation to visual stimuli - including written words, false font strings, faces, houses, and objects - at both time points. Our primary aim was to determine whether diminished neural adaptation is present before extensive reading experience, which would support its role as a core deficit. Alternatively, if neural adaptation differences emerge only after formal reading instruction, this would suggest that adaptation is influenced by experience with written language. We also investigated whether adaptation deficits are domain-specific, particularly for word stimuli in the left ventral occipito-temporal cortex, or domain-general (Perrachione et al., 2016), and whether individual differences in adaptation relate to core pre-reading skills.

## 2 Materials and methods

### 2.1 Participants

Participants came from a larger longitudinal study examining literacy development (see Chyl et al., 2023, Dębska et al., 2024). Children underwent an initial screening session, completed four in-lab testing sessions conducted at 12-month intervals, including behavioral assessments and an fMRI-RA scan. We refer to the first experimental session, conducted in kindergarten, as time-point 1 (TP1), and the last experimental session, conducted in the 3rd grade of primary school, as TP2. Because the study took place during COVID-19 pandemic, attendance for the other two sessions was much lower.

Children were included if they met following criteria: (1) birth at term (>37 weeks), right-handedness, monolingualism, normal or corrected-to-normal vision, no history of neurological illness or brain damage, no reported ADHD symptoms, no contraindications to MRI, being a pre-reader at initial screening; (2) for cross-sectional analyses, complete data for all behavioral tasks and fMRI-RA data at TP1; (3) for longitudinal analyses, complete measurements of word- and pseudoword reading tests and fMRI-RA data at TP1 and TP2; (4) at least 3 out of 4 runs in the fMRI-RA task at each TP meeting movement criteria.

Participants with reading difficulties were assigned to the dyslexic group based on the clinical dyslexia diagnosis performed in 3rd grade by clinical child psychologists in psychological and pedagogical counseling centers. Two participants with reading difficulties did not undergo a clinical diagnosis of dyslexia, but were assigned to the dyslexic group based on their low word and pseudoword reading scores (below 2 SD in the second grade and/or lower than 1.5 SD from the mean of the control group in the first grade).

Out of 56 participants initially included in the longitudinal dataset after checking the completeness of fMRI data, 47 children who met movement criteria during the fMRI-RA scan were retained for the analyses. Although all children were recruited as pre-readers during kindergarten in Poland and had not yet received formal reading instruction, some had developed early reading skills by the time of the fMRI scan. This resulted in significant group differences in reading performance at TP1 (see Supplementary Materials 3.1.1)*.* Therefore, we used case-control matching in SPSS to match the dyslexic and control groups in terms of age and word reading score at TP1 (see Supplementary Materials 1.1.). This resulted in 16 matched pairs – groups did not differ significantly in sex (χ^2^(1, N = 32) = 0.51, *p* = 0.48; control group included 8 boys and 8 girls, dyslexic group 10 boys and 6 girls), age at TP1 (CON=6.79 ± 0.34; DYS=6.57 ± 0.35); *t* = 1.77, *p* = 0.087), and TP2 (CON =9.91 ± 0.27; DYS=9.69 ± 0.37); *t* = 1.86, *p* = 0.073). We report the results for the matched sample in the main text, and the results for the entire longitudinal sample (25 control and 22 dyslexic children) in Supplementary Materials 3.1.

Fifty-nine out of 68 participants scanned at TP1 met the movement criteria during the fMRI-RA scan and were included in the individual differences analyses examining the association between neural adaptation at TP1 and core pre-reading skills (see Table S3 in Supplementary Materials for demographic and behavioral scores).

The study was approved by the Research Ethics Committee at the SWPS University of Social Sciences and Humanities in Warsaw (date of approval 13/10/2015, approval number 28/2015). Parents of children who participated signed an informed consent form, and children agreed verbally. Families were reimbursed for participation in the project.

The study hypotheses and analytical plan were pre-registered through Open Science Framework prior to analyses (https://osf.io/5wmvq), but were modified following suggestions from anonymous reviewers (see Supplementary Materials 1.1.– 1.5.).

### 2.2 Method details

#### 2.2.1 Behavioral tests

The following behavioral tests were collected at TP1. Non-verbal IQ was assessed with the Cattell Culture Fair Intelligence Test (CFT 1-R; Koć-Januchta et al., 2013). Phonological awareness (PA) was examined with three tasks: standardized test of phoneme analysis (Szczerbiński and Pelc-Pękala, 2013), and two in-house tests of alliteration and rhyming. In the alliteration task, children were asked to select one word out of three that started with the same phoneme as the target word. Eighteen target items (real words) were read aloud by the experimenter. The correct answer given immediately was worth 2 points, and the correct answer given after stimuli repetition - 1 point. In the rhyming task, children were asked to select one word out of three that rhymed with the target word. Letter knowledge containing upper and lower cases was examined with a standardized test (Szczerbiński and Pelc-Pękala, 2013). Rapid automatized naming (RAN) was tested with object and color naming subtests (Fecenec et al., 2013). The score represents a reaction time required to name all the items. Visual attention was tested with a subtest of the IDS Intelligence Scale measuring visual selective attention (Jaworowska et al., 2012).

For the assessment of reading skill over time, word and pseudoword reading efficiency was measured (Szczerbiński and Pelc-Pękala, 2013) each year on the same day as an fMRI session. In the word reading subtest, children were asked to read aloud as many words as possible in 30 s. Two parallel sheets of 75 words increasing in length were provided, starting with two 2-letter words, next five 3-letter words, six 4-letter words and so on. The total number of correctly read words in a minute (the sum of the two lists) was calculated. Pseudoword reading efficiency (Szczerbiński and Pelc-Pękala, 2013) had an analogous procedure.

#### 2.2.2 Data acquisition

The fMRI data were acquired on a 3-T Siemens Trio scanner using a whole-brain EPI sequence with a 32-channel head coil, 32 transverse slices, 3.5 mm slice thickness, 2 s TR, 30 ms echo time, 90° flip angle, 192 mm^2^ FOV, voxel resolution = 3.5 mm^3^, 64 × 64 matrix. Anatomical data were acquired with a T1-weighted sequence with 176 slices, 1 mm slice thickness, 2.53 s TR, 3.34 ms TE, 7° flip angle, 256 × 256 matrix, voxel size = 1 mm^3^.

#### 2.2.3 fMRI-RA task and procedure

Children were introduced to the task in a mock scanner. The task consisted of four runs, each lasting 5.13 min (20.5 min in total).

**Fig. 1:**
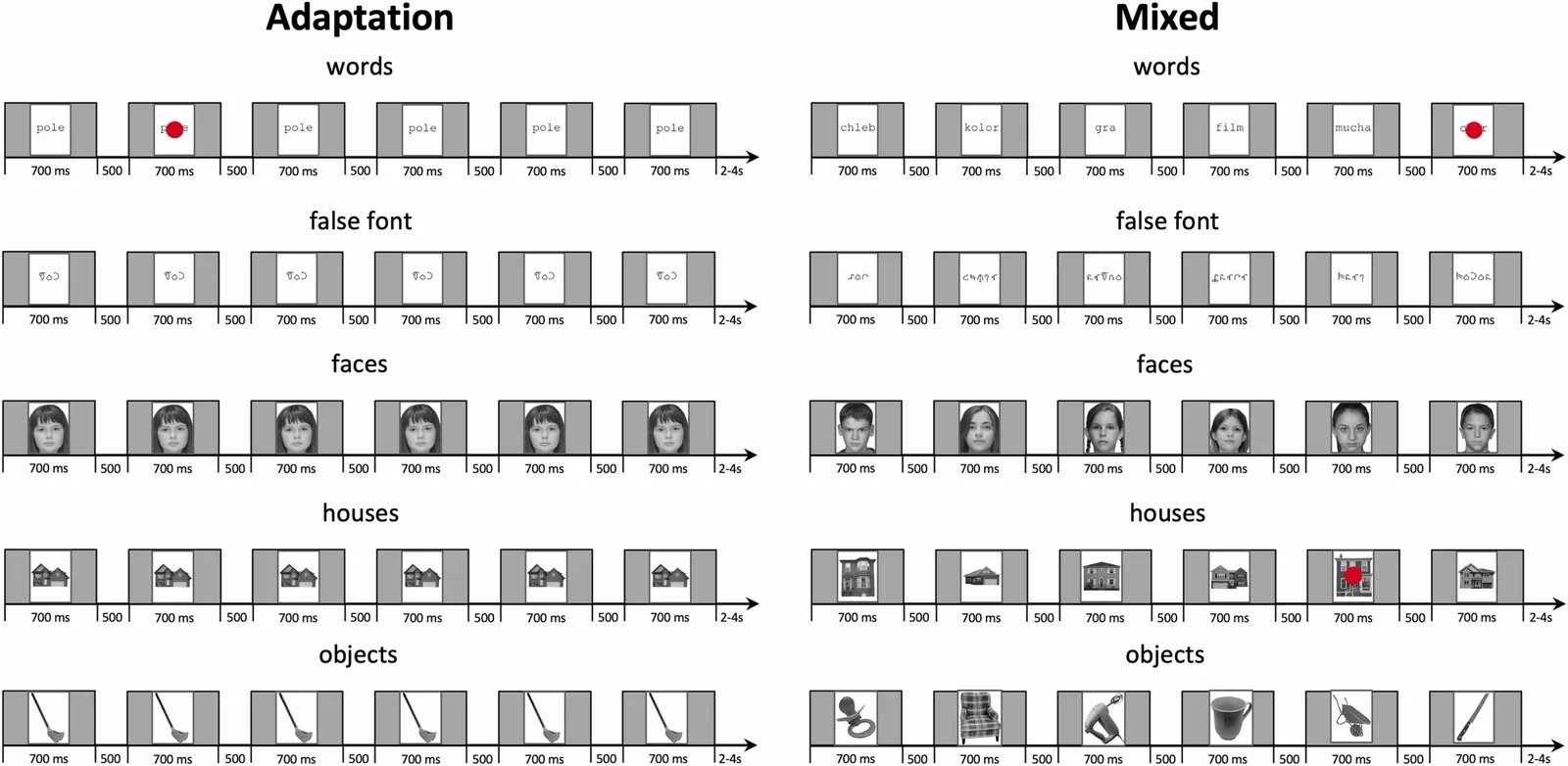
Schematic of the fMRI stimulation paradigm. Five different categories of stimuli were used: (1) high-frequency Polish nouns from the Childes Corpus in Courier font, (2) the same words in the BACS false font (Vidal et al., 2017), (3) neutral children's faces (Meuwissen et al., 2017), (4) houses dissimilar to faces (Filliter et al., 2016), and (5) familiar everyday objects (Brodeur et al., 2014). Neurophysiological adaptation was measured by contrasting blocks of viewing multiple different stimuli (mixed) versus blocks with the repeated presentation of a single stimulus (adaptation). Targets (red dots) appeared in 10/30 blocks in each run, in any position but first.

The stimuli were presented in mini-blocks containing six items, each was displayed for 700 ms, followed by a 500 ms interstimulus interval. Five different categories of stimuli were used: (1) 3–5 letter, high-frequency Polish nouns from the Childes Corpus in Courier font, (2) the same words in the BACS false font (Vidal et al., 2017), (3) neutral children's faces (Meuwissen et al., 2017), (4) houses dissimilar to faces (Filliter et al., 2016), and (5) familiar everyday objects (Brodeur et al., 2014). The Shine toolbox (Willenbockel et al., 2010) was used to equalize luminance and contrast across the stimuli. After each block, a blank screen was shown for 1–3 s (see Fig. 1). Each run consisted of 30 mini-blocks. In Adaptation (ADAPT) blocks, the same image was repeated six times, while in Mixed (MIX) blocks, six different items from the same category were presented. In 10 out of the 30 blocks in each run, a red target dot appeared on the screen in any position within the block except the first. Children were asked to press the left button on the response pad whenever a target appeared on an image. Groups did not differ in the number of correct responses to target trials or reaction times (see Supplementary Materials 2.1.2 and 2.2.2). The block types and target occurrences across categories were balanced within the run. The experiment was programmed using Presentation software (Neurobehavioral Systems, Albany, CA).

### 2.3 Statistical analysis

#### 2.3.1 fMRI data preprocessing

The preprocessing and statistical analyses were performed using SPM12 (Wellcome Imaging Department, UCL, UK, http://fil.ion.ucl.ac.uk/spm), run in MATLAB R2024a (The MathWorks Inc. Natick, MA, USA). For longitudinal analysis, T1w images were bias-corrected and registered to a within-subject average (avgT1w) using Pairwise Longitudinal Registration (Ashburner and Ridgway, 2013). AvgT1w (longitudinal) and T1w (cross-sectional) images were coregistered to the mean functional image, segmented with pediatric tissue maps (Wilke et al., 2008), and normalized. Normalization to the MNI space was carried out using deformation fields acquired from avgT1w/T1w images, with voxel size 2 × 2 × 2 mm. Normalized images were smoothed with an 8 mm FWHM Gaussian kernel. Volumes exceeding 3 mm and a rotation threshold of 0.05 radians were identified as artifactual using ART Toolbox (https://www.nitrc.org/projects/artifact_detect). Runs with more than 10% of volumes exceeding this threshold were excluded (see Supplementary Materials 1.2). For the remaining runs, ArtRepair (Mazaika et al., 2007) was used to identify and repair motion-affected outlier volumes using interpolated values of the adjacent non-outlier volumes. Outlier volumes were defined as volumes exceeding 1.5 mm volume-to-volume head movement, total head movement greater than 5 mm, or more than 4% of variations in the global mean intensity. Groups did not differ in the number of retained runs or censored volumes at both TPs (see Supplementary Materials 2.1.1 and 2.2.1).

#### 2.3.2 Statistical analysis – behavioral measurements

Behavioral analyses were conducted using R version 4.4.1 (R Core Team, 2021) and SPSS version 29.0.2.0. For word and pseudoword reading, two-way repeated measures ANOVAs were performed with group as a between-subject factor and time point as a within-subject factor using the *afex* package in R (Singmann et al., 2024). Post-hoc pairwise comparisons were conducted using the emmeans package (Lenth, 2025) and corrected for multiple comparisons using Bonferroni method. Additionally, we examined group differences in age, IQ, RAN (average time to complete the objects and colors subtests), PA (a principal component comprising phoneme analysis, alliteration, and rhyming), letter knowledge, and visual attention using independent samples *t*-tests. Results for the entire longitudinal sample are reported in Supplementary Materials 3.1.1.

### 2.4 Statistical analysis – task-based activation

Statistical analyses performed at the individual and group level were run with code adapted from Chyl et al. (2023), published online by the authors (https://osf.io/tp5uf/). A copy of the exact codes used for this project can be found here: https://osf.io/ytfcv.

#### 2.4.1 First-level (individual) analysis

Repaired volumes in all runs were entered into general linear models. Timings of experimental blocks and fixation periods were modelled as regressors of interest, with six motion parameters estimated during realignment, composite motion parameters and artifactual volumes defined by ART Toolbox (Siegel et al., 2014) entered as regressors of no interest. We also entered timings of oddball red dot trials together with the following interstimuli intervals (altogether 1200 ms for each target trial) as additional regressors of no interest (for more details see Supplementary Materials 1.3). Data were high-pass filtered with a cut-off period of 1/128 Hz with SPM mask threshold of 0.5 based on previous studies of 5–10-year-old children (e.g. Weiss et al., 2018; Wang et al., 2021; Wang et al., 2023). Separate first-level models were computed for longitudinal and cross-sectional samples. To examine neural adaptation effects for each category, contrasts of *category_mix > category_adapt* (e.g. *Faces_mix > Faces_adapt*) were computed for each TP. Contrasts representing the average of both TPs – namely, *category_mix > category_adapt* over TP1 and TP2 – were specified to obtain the main effect of the group in the longitudinal analysis. Contrasts *category_mix > baseline* were computed to define regions of interest (ROIs; see *ROI analysis* section for further information).

#### 2.4.2 Second-level (group) analysis

To align with current best practices (e.g., Eklund et al., 2016), we report all results using a voxel-wise threshold of p < 0.001, combined with a cluster-wise p < 0.05 family-wise error correction (FWEc). These results were complemented with the pre-registered FDRc correction, if FWE and FDR cluster extents differed. For details, see Supplementary Materials 1.5.

Furthermore, group-level results were masked with a combined positive activation image. First-level contrasts (category_mix + category_adapt) at each TP were entered into one-sample *t*-tests across all participants (voxel-level p < 0.05), binarized, and summed using ImCalc to create the final mask. Details of the voxel-wise threshold and rationale for a positive activation mask are provided in the Supplementary Materials 1.5.

*Longitudinal analysis*: one-sample *t*-tests were performed to examine adaptation effects for each category in each group at each TP. To assess group x TP interaction, a flexible 2 × 2 factorial model with group and TP - specified with unequal variance - and subject factor with equal variance, was used. A paired *t*-test was performed to compare changes in time for both groups pooled together. To obtain the main effect of the group, a two-sample *t*-test was performed comparing the average brain activation across TPs. Results for the entire longitudinal sample are reported in Supplementary Materials 3.1.2.

*TP1 analysis:* one-sample *t*-tests were performed to examine adaptation effects for each category in the entire sample (for results, see Supplementary Materials 4).

#### 2.4.3 ROI analysis

We performed non-preregistered exploratory two-way ANOVAs with a within-subject factor of TP and a between-subject factor of group for adaptation to words in the bilateral vOT, adaptation to faces in the bilateral FFA, and adaptation to objects in the bilateral LOC. These ROIs were defined either at the group-level or individually (see below and Supplementary Materials 1.4.). ROI analyses with the entire longitudinal sample are reported in Supplementary Materials 3.1.3.

We also examined the association between individual differences in adaptation and pre-reading skills in the entire sample tested at TP1. First, we regressed IQ and age out of the behavioral measures (PA, letter knowledge, RAN, visual attention, word, and pseudoword reading), and then we correlated the standardized residuals with adaptation in the selected ROIs using Spearman’s correlations (see section “Cross-sectional analysis: brain-behavior correlations at TP1” below).

#### 2.4.4 Group-defined ROIs

Beta values were extracted from three regions per hemisphere: the ventral occipito-temporal cortex (vOT), fusiform face area (FFA), and lateral occipital cortex (LOC). Left vOT was defined based on the intersection of an independent mask from Dębska et al. (2026) and the overall activation mask of *Words_mix > baseline* from the total sample (at p < 0.001 uncorrected). Then, we created its right-hemispheric homologue using the MATLAB *flip* function (see Supplementary Materials 1.4.1.). The bilateral vOT ROI was then used for the contrast *Words_mix* > *Words_adapt*. FFA was based on the face-selective activation [FFA: −39, −45, −18] (Downing et al., 2006, Feng et al., 2020), built as a 7 mm sphere and its right-hemispheric homologue, and masked with the overall activation mask of *Faces_mix > baseline* from the total sample (at p < 0.001 uncorrected) and used for the contrast *Faces_mix > Faces_adapt* (see Chyl et al., 2023). Bilateral lateral occipital cortex (LOC) was defined as an intersection of anatomical masks of the left and right LOC (using the Harvard-Oxford atlas, https://cma.mgh.harvard.edu/) and the overall activation mask of *Objects_mix > baseline* from the total sample (at p < 0.001 uncorrected), used for the contrast *Objects_mix > Objects_adapt*. Since the neural adaptation effects for words were not present in the left IFG and left posterior temporal cortex, we did not include these pre-registered ROIs in the analyses.

#### 2.4.5 Individually-defined ROIs

Since individual variability in the location of word- or face-selective cortex may be obscured by averaging responses within a group-defined ROI, we performed a complementary ROI analysis using individually-defined functional ROIs (for details see Supplementary Materials 1.4.2.). First, we defined a common left and right vOT search space based on the group-level *Words_mix > baseline* contrast averaged across TP1 and TP2, while excluding the cerebellum (using all cerebellum anatomical masks from the AAL3 atlas (Rolls et al., 2020) combined in ImCalc). This search space showed a bimodal posterior–anterior voxel distribution, therefore, we decided to divide this search space to more anterior regions (y > -70) and more posterior regions (y < -70; see Figure S2). Within these two search spaces per hemisphere, participant-specific word-selective ROIs were defined using the *Words_mix > Houses_mix* contrast averaged across TP1 and TP2. Voxels were ranked according to their positive t-values, and up to the 100 strongest positive voxels were retained for each participant. Next, word adaptation, defined as *Words_mix > Words_adapt*, was extracted separately for TP1 and TP2 from the same participant-specific ROI. Analogical approach was used to define face and object selective cortex. Because the corresponding face- and object-responsive search spaces extended beyond the regions of interest, the *Faces_mix > baseline* map was additionally constrained by the left and right fusiform-gyrus masks from the AAL3 atlas, while the *Objects_mix > baseline* map was constrained by the left and right LOC masks from Harvard-Oxford atlas. Within each search space, participant-specific ROIs were defined as the 100 voxels showing the strongest category-selective response (i.e., *Faces_mix > Houses_mix* and *Objects_mix > Houses_mix*), averaged across TP1 and TP2. Next, face adaptation, defined as *Faces_mix > Faces_adapt* as well as object adaptation (*Objects_mix > Objects_adapt*) were extracted separately for TP1 and TP2 from participant-specific ROI. When fewer than 100 voxels were available, all eligible voxels were retained, and the resulting ROI size was recorded (see Figure S3 for the overlap between individual ROIs).

For cross-sectional sample, we used the same search spaces as for longitudinal sample, but participant-specific ROIs were defined as the 100 voxels showing the strongest category-selective response (*Words_mix > Houses_mix*, *Faces_mix > Houses_mix* and *Objects_mix > Houses_mix*) at TP1.

## 3 Results

### 3.1 Behavioral results

The matched groups did not differ significantly in IQ or pre-reading skills measured at TP1 (see Table 1).

**Table 1:** Test performance during TP1 (kindergarten) in the control and dyslexic group (N = 16 participants per group).

	Control			Dyslexic			Independent samples test	
	Mean	SD	Range	Mean	SD	Range	t	p
Cattell IQ	105.75	13.39	77–120	110.19	13.26	88–138	-0.94	0.354
PA	0.10	0.91	-1.72–1.59	-0.41	0.93	-1.72–1.64	1.59	0.123
RAN (sec)	83.06	21.97	53.5–140	96.19	21.06	63–128.5	-1.73	0.095
Letter knowledge (upper & lower case)	44.69	13.38	20–62	35.69	15.80	10–63	1.85	0.074
Visual Attention (max score 105)	36.13	8.27	20–52	36.06	9.47	15–54	0.02	0.984

**Fig. 2:**
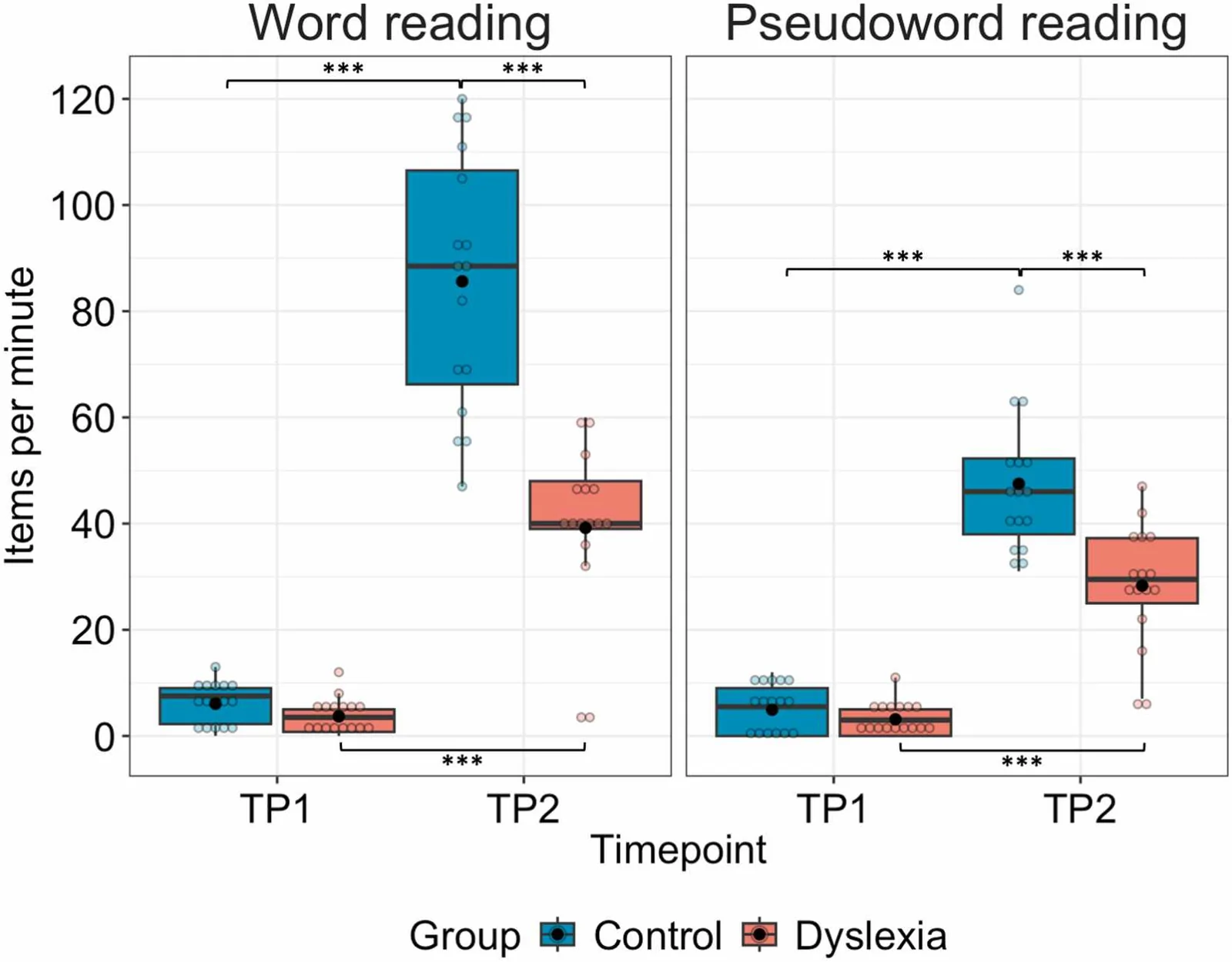
Word and pseudoword reading performance in the control and dyslexic group at two time points (TP; N = 16 participants per group). Black dots indicate mean values. Significant differences between groups and TPs are indicated with asterisks, *** *p* ≤ 0.001.

For word reading, there were significant main effects of group (*F*(1,30) = 40.35, *p* < 0.001, η_p_^2^ = 0.57), TP (*F*(1,30) = 271.49, *p* < 0.001, η_p_^2^ = 0.90), and interaction of group x TP (*F*(1,30) = 39.81, *p* < 0.001, η_p_^2^ = 0.57). For pseudoword reading analysis showed analogous results: a main effect of the group (*F*(1,30) = 19.46, *p* < 0.001, η_p_^2^ = 0.39), TP (*F*(1,30) = 220.31, *p* < 0.001, η_p_^2^ = 0.88), and interaction of group x TP (*F*(1,30) = 14.49, *p* < 0.001, η_p_^2^ = 0.33). The post-hoc analysis revealed that groups did not differ in word reading at TP1 (*t*(30) = 1.75, *p* = 0.539; *M* dyslexic group = 3.7, *M* control group = 6.1). However, control children read significantly more words than dyslexic children at TP2 (*t*(30) = 6.44, *p* < 0.001; *M* dyslexic group = 39.2, *M* control group = 85.6). Both groups showed a significant improvement in word reading from TP1 to TP2 (control group: *t*(30) = 16.11, *p* < 0.001; dyslexic group: *t*(30) = 7.19, *p* < 0.001), though it was more pronounced in controls. Similarly, for the pseudoword reading, control children performed significantly better compared to dyslexic children at TP2 (*t*(30) = 4.29, *p* = 0.001; *M* dyslexic group = 28.3, *M* control group = 47.5), but not at TP1 (*t*(30) = 1.39, *p* = 1.00; *M* dyslexic group = 3.1, *M* control group = 5.0). Both groups improved significantly in pseudoword reading from TP1 to TP2 (control group: *t*(30) = 13.19, *p* < 0.001; dyslexic group: *t*(30) = 7.80, *p* < 0.001), but again controls showed larger progress (see Fig. 2).

#### 3.1.1 Whole-brain fMRI results

**Fig. 3:**
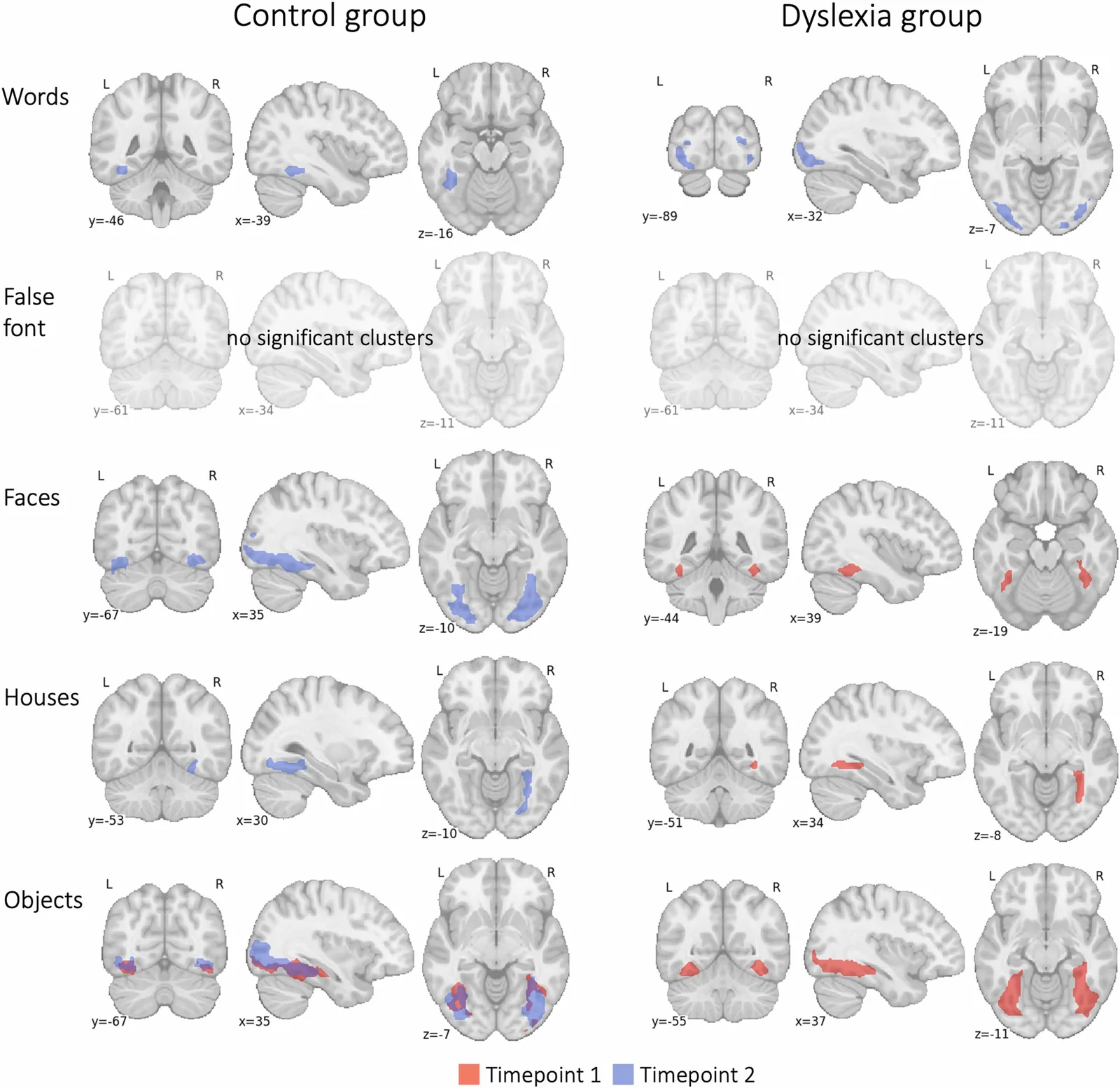
Adaptation to visual stimuli categories in control children and children with and without dyslexia at TP1 and TP2 (N = 16 participants per group). All contrasts at voxelwise *p* < 0.001 and clusterwise *p* < 0.05 FWE corrected. The only exception is the contrast for words in the control group at TP2, which is presented at voxelwise *p* < 0.001 and clusterwise *p* < 0.05 FDR corrected, but did not survive a voxelwise *p* < 0.001 and clusterwise *p* < 0.05 FWE correction.

In controls, word adaptation appeared at TP2 in the left ventral occipito-temporal cortex. Note, however, that this result was significant only after the clusterwise FDR correction, but did not reach significance using clusterwise FWE correction. In dyslexia, word adaptation was seen only at TP2 in the bilateral inferior and middle occipital cortex. Neither group showed adaptation to false font strings. Face adaptation was found at TP2 in controls (bilateral fusiform, occipital, right lingual gyrus, left cerebellum) and at TP1 in dyslexia (bilateral fusiform and right temporal gyri). House adaptation was present at TP2 in controls in the right hemisphere in the fusiform and lingual gyri and at TP1 in the dyslexic group in a similar area. Object adaptation occurred in controls at both TPs (bilateral fusiform, inferior occipito-temporal gyri, left cerebellum) and in dyslexia at TP1 (bilateral fusiform, occipito-temporal and parahippocampal gyri). For details, see Fig. 3 and Table 2.

**Table 2:** Neurophysiological adaptation to different categories of stimuli in control and dyslexic children at TP1 (kindergarten) and TP2 (3rd grade; N = 16 participants per group). Significant effects at voxelwise p < 0.001, and clusterwise *p* < 0.05 FWE corrected. The only exception is the result for words in the control group at TP2, which is presented at voxelwise *p* < 0.001 and clusterwise *p* < 0.05 FDR corrected.

				MNI Coordinates		
Contrast Name	Region Label	Extent	t-value	x	y	z
**TP1**						
Control group: objects^a^	Fusiform_R, Occipital_Inf_R, Temporal_Inf_R, ParaHippocampal_R	1860	8.68	30	-36	-20
	Fusiform_L, Temporal_Inf_L, Occipital_Inf_L, Cerebelum_6_L	1892	9.02	-36	-38	-18
Dyslexic group: faces^b^	Fusiform_R	252	7.55	40	-46	-22
	Fusiform_L, Temporal_Inf_L	238	6.89	-36	-38	-18
Dyslexic group: houses^c^	Fusiform R	250	7.66	36	-50	-8
Dyslexic group: objects^d^	Fusiform R, Occipital_Inf_R, Temporal_Inf_R, ParaHippocampal_R	1745	7.57	32	-38	-14
	Fusiform L, Occipital_Inf_L,	1685	8.13	-34	-78	-12
	ParaHippocampal_L					
**TP2**						
Control group: words^e^	Fusiform_L, Temporal_Inf_L	225	6.25	-40	-44	-18
Control group: faces^f^	Fusiform_R, Occipital_Inf_R, Occipital_Mid_R, Lingual_R, Calcarine_R	2203	8.63	30	-98	0
	Fusiform_L, Occipital_Inf_L, Occipital_Mid_L, Cerebelum_6_L	1626	6.14	-34	-40	-20
Control group: houses^g^	Fusiform_R, Lingual_R	508	9.37	32	-58	-8
Control group: objects^h^	Fusiform_R, Occipital_Mid_R, Occipital_Inf_R, Temporal_Inf_R	2260	8.98	34	-42	-16
	Occipital_Mid_L, Fusiform_L, Occipital_Inf_L	1854	8.65	-32	-54	-18
Dyslexic group: words^i^	Occipital_Inf_R, Occipital_Mid_R, Fusiform_R, Temporal_Inf_R, Lingual_R	761	7.00	-42	-82	-12
	Occipital_Inf_L, Occipital_Mid_L, Lingual_L, Fusiform_L	825	8.62	-38	-88	-10

Note: Regions were automatically labeled using the AAL3 with a minimum contribution of 5% to the clusters, x, y, and z Montreal Neurological Institute (MNI) coordinates in the left-right, anterior-posterior, and inferior-superior dimensions, respectively. ^a^FDR cluster extent = 1892, ^b^FDR cluster extent = 252, ^c^FDR cluster extent=not significant, ^d^FDR cluster extent=not significant, ^e^FDR cluster extent = 225; FWE cluster extent=not significant, ^f^FDR cluster extent = 2203, ^g^FDR cluster extent=not significant, ^h^FDR cluster extent = 2260, ^i^FDR cluster extent = 825.

No significant main effects of group, time point, or group × TP interaction was found.

### 3.2 ROI results

#### 3.2.1 Group-defined ROIs

**Fig. 4:**
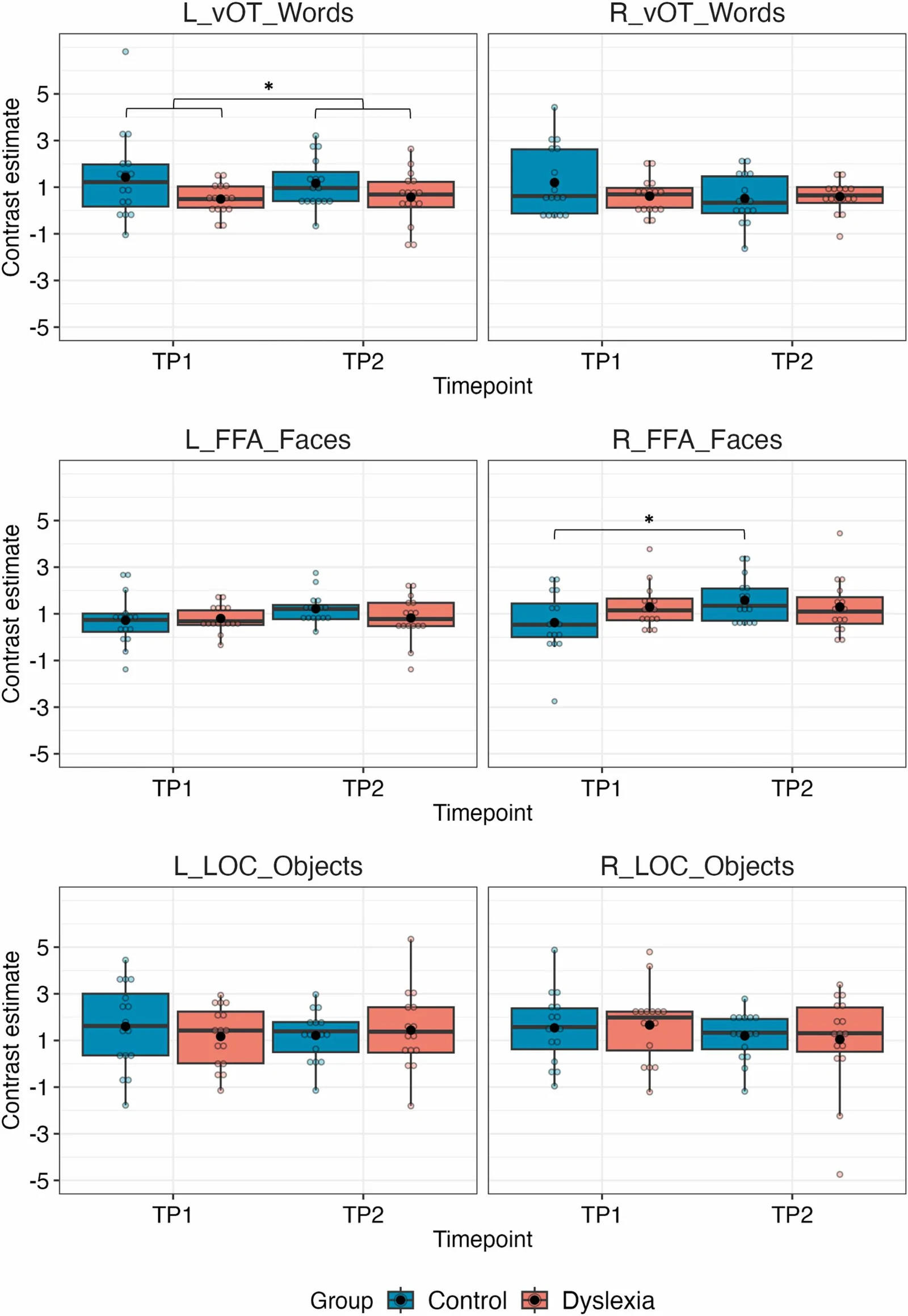
Results for group-defined ROI analyses for matched participants (N = 16 participants per group): adaptation to words in the bilateral vOT, adaptation to faces in the bilateral FFA, and adaptation to objects in the bilateral LOC. A significant main effect of the group in the left vOT and a significant difference between TPs within the control group in the right FFA are indicated with asterisks, * *p* < 0.05.

Results revealed significant effects only in the case of adaptation to words in the left vOT and adaptation to faces in the right FFA. For the left vOT a significant effect of group (*F*(1,30) = 7.07, *p* = 0.012, η_p_^2^ = 0.19) was found with stronger overall adaptation to words in the control than the dyslexic group, while the effect of time (*F*(1,30) = 0.07, *p* = 0.794) and time x group interaction (*F*(1,30) = 0.25, *p* = 0.621) were not significant. In the right FFA, a significant effect of time (*F*(1,30) = 4.32, *p* = 0.046, η_p_^2^ = 0.13), with stronger adaptation to faces at TP2 compared to TP1, and group x time interaction (*F*(1,30) = 4.37, *p* = 0.045, η_p_^2^ = 0.13) were observed. Post-hoc analyses revealed that only the control group presented a significant increase of the neural adaptation with time (*t*(30) = 2.95, *p* = 0.037), while it was not present in the dyslexic group (*t*(30) = 0.01, *p* = 1.0). See Fig. 4 for details.

#### 3.2.2 Individually-defined ROIs

**Fig. 5:**
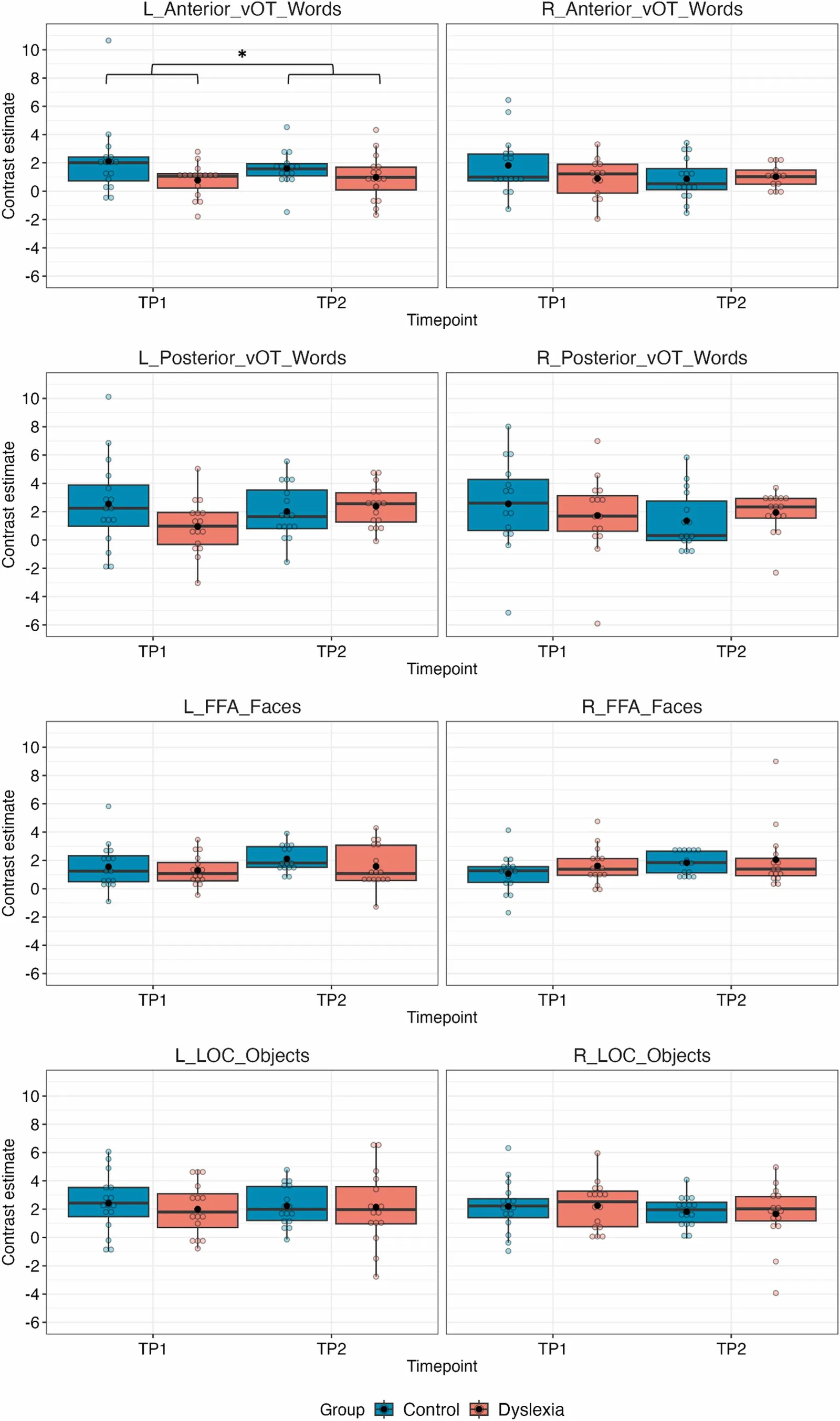
Results for individually-defined ROI analyses for matched participants (N = 16 participants per group): adaptation to words in the bilateral anterior and posterior vOT, adaptation to faces in the bilateral FFA, and adaptation to objects in the bilateral LOC. A significant main effect of the group in the left anterior vOT is indicated with asterisks, * *p* < 0.05.

Results revealed a significant effect only in the case of adaptation to words in the left anterior vOT. Namely, we observed a main effect of group, *F*(1, 30) = 4.71, *p* = .038, ηp² = .136, with stronger adaptation to words in the control than the dyslexic group. There was no significant effect of time point, *F*(1, 30) = 0.14, *p* = .708, ηp² = .005, nor the group × time point interaction, *F*(1, 30) = 0.72, *p* = .403, ηp² = .023. No significant effects were observed in the left posterior vOT, in the right anterior or posterior vOT, bilateral FFA and LOC. See Fig. 5 for details.

#### 3.2.3 Cross-sectional analysis: brain-behavior correlations at TP1

We examined associations between adaptation in all selected group- and individually-defined ROIs and pre-reading skills across the entire sample at TP1 (controlling for IQ or age), using Spearman’s correlation. For group-defined ROIs word adaptation in left vOT correlated positively with PA as well as words- and pseudowords read per minute (Table 3, Figure S9B). Face adaptation in the right FFA correlated negatively with PA. For individually-defined ROIs, results revealed a positive relationship only between word adaptation in the left anterior vOT and word-reading (Table 4, Figure S9C). Although all correlations reached nominal significance (p < 0.05), none survived correction for multiple comparisons using the Benjamini–Hochberg false discovery rate (FDR) procedure.

**Table 3:** Spearman’s correlations between pre-reading skills and group-defined ROIs (N = 59 participants).

Variable	PA	RAN	Visual attention	Letters	Word Reading	Pseudoword Reading
Words_L_VOT	0.29*	-0.14	0.2	0.24	0.33*	0.29*
Words_R_VOT	0.23	0.01	0.08	0.03	0.26	0.22
Faces_L_FFA	-0.14	0.09	-0.07	-0.19	-0.23	-0.18
Faces_R_FFA	-0.27*	0.07	-0.14	-0.01	-0.06	0
Objects_L_LOC	-0.07	-0.11	0.04	0.01	-0.05	-0.08
Objects_R_LOC	-0.2	-0.01	-0.03	-0.03	-0.08	-0.1

Note: *p < 0.05 (two-tailed uncorrected).

**Table 4:** Spearman’s correlations between pre-reading skills and individually-defined ROIs (N = 59 participants).

Variable	PA	RAN	Visual attention	Letters	Word Reading	Pseudoword Reading
Words_L_aVOT	0.17	-0.07	0.11	0.16	0.28*	0.24
Words_L_pVOT	0.17	-0.02	0.22	0.12	0.07	0.05
Words_R_aVOT	0.11	0.03	0.16	0.12	0.22	0.21
Words_R_pVOT	0.22	-0.14	0.14	0.23	0.10	0.04
Faces_L_FFA	-0.05	0.13	-0.16	-0.02	-0.13	-0.09
Faces_R_FFA	-0.22	0.04	-0.19	-0.16	-0.09	-0.04
Objects_L_LOC	0.16	-0.01	0.08	-0.20	-0.01	0.01
Objects_R_LOC	0.23	0.08	0.14	-0.09	-0.06	-0.04

Note: *p < 0.05 (two-tailed uncorrected).

In line with preregistration, we also performed correlations between pre-reading skills and neural adaptation in all ROIs in control and dyslexic groups separately. Results are reported in Supplementary Materials, Tables S6-S9.

## 4 Discussion

The present longitudinal fMRI study investigated whether altered neural adaptation (Perrachione et al., 2016) constitutes a precursor of dyslexia or emerges as a consequence of reading experience. By following children oversampled for dyslexia risk from kindergarten to third grade and applying a rapid adaptation paradigm across linguistic and non-linguistic visual categories, we aimed to clarify the developmental timing, specificity, and functional relevance of neural adaptation differences. All primary analyses were preregistered (OSF: https://osf.io/5wmvq), with several deviations reported in the Supplementary Materials.

Consistent with our hypothesis, children who later developed dyslexia showed reduced neural adaptation to written words in the left vOT ROI already at kindergarten, when groups were matched on reading (and other pre-reading) skills through case-control matching. These findings were supported by both group- and individually-defined ROI analyses. The observed difference remained stable across development, with no significant time or group × time interaction, suggesting that reduced word-related adaptation in the left vOT may reflect an early-emerging neurofunctional difference associated with later dyslexia, rather than arising solely as a consequence of reduced reading exposure. Importantly, after accounting for inter-individual variability in the location of category-selective cortex by defining ROIs individually, we found that the observed effects were restricted to the left anterior vOT, corresponding with the anterior part of the visual word form area (VWFA-2, Yablonski et al., 2024), and middle occipito-temporal sulcus (Lerma-Usabiaga et al., 2018). No such effects were present in the posterior vOT. Formal reading instruction is not a part of curriculum in Polish kindergartens. Nevertheless, some variation in early word reading appeared, children knew most letters in the alphabet and some of them could read several sight words per minute. This suggests that the left anterior vOT sensitivity to repeated word stimuli emerges in typically reading children even before extensive reading experience (Brem et al., 2010), and is in line with letter-specific EEG responses in preschoolers with letter knowledge (Lochy et al., 2016). Importantly, the group difference, but no significant time or group × time interaction, was also observed in the entire longitudinal sample (see Supplementary Materials 3.1.3), which differed in early reading and pre-reading skills at TP1.

Across the full kindergarten sample, greater adaptation to words in the left vOT was positively associated with both early reading ability (in both group- and individually defined ROIs) and phonological awareness (in group-defined ROI), consistent with prior evidence that individual differences in ventral visual tuning for print are continuously related to emerging literacy skills (e.g., Maurer et al., 2006; Brem et al., 2010; Centanni et al., 2018; Dehaene-Lambertz et al., 2018). Positive association with phonological awareness aligns with the reciprocal phonology-orthography development (Dębska et al., 2026, Landerl et al., 2019). Children who are better at distinguishing the sounds of words also tend to recognize graphemes more easily, which leads to faster adaptation to visual word forms. In turn, stronger visual word adaptation, reflecting more robust orthographic selectivity (Glezer et al., 2016), appears to support more efficient meta-phonological processing. Indeed, word adaptation in the left vOT (defined at both group- and individual level) showed a positive correlation with the phonological awareness skill in the control group, but not in children with dyslexia. These results extend previous work in adults and literate children (Perrachione et al., 2016), showing that word-specific adaptation differences associated with later dyslexia can be detected prior to formal reading instruction and vary with pre-reading skills, supporting the view that atypical tuning of print-selective cortex may contribute to reading difficulties rather than merely reflecting their downstream effects. At the same time, the modest effect sizes and absence of whole-brain group differences underscore that such differences are subtle and best understood within a dimensional framework rather than as a categorical neural deficit.

Contrary to the domain-general adaptation deficits reported previously (Perrachione et al., 2016), we found no evidence for reduced neural adaptation in dyslexia across non-linguistic visual categories at the whole-brain level. Our study employed more stringent statistical thresholds (voxel-wise *p* < 0.001, cluster-corrected), in contrast to the more liberal threshold (*p* < 0.05, cluster-corrected) used in the original study. Notably, such liberal thresholds have been shown to inflate false positive rates (Eklund et al., 2016), calling into question the robustness of the previously reported group differences. In the current study, both groups exhibited adaptation to faces, houses, and objects, with no robust group differences in either whole-brain or preregistered ROI analyses. However, given the modest sample size, the whole-brain findings should be interpreted with caution. These findings align with recent studies reporting intact auditory and visual adaptation in adults with dyslexia (Beach et al., 2022, Tan et al., 2022) and suggest that previously reported domain-general deficits may depend on task, stimulus complexity, or analytical thresholds. Together, these results provide little support for a pervasive deficit in repetition-based neural tuning and instead point to more stimulus- and region-specific alterations.

We did not observe reliable neural adaptation to false font strings in either group at the whole-brain level. It emerged only when analyses were conducted in the larger cross-sectional sample at TP1, indicating that repetition-related responses to visually unfamiliar, non-linguistic stimuli were relatively weak and variable across individuals. False font strings lack stable orthographic and semantic associations and therefore may fail to engage well-established visual representations capable of supporting robust repetition suppression in high-level visual cortex (Glezer et al., 2009). These findings suggest that neural adaptation in ventral visual regions may be shaped by experience-dependent tuning to meaningful visual categories rather than by visual repetition per se. Consistent with prior work, neural tuning appears to depend on both cumulative visual experience with a stimulus class (Grill-Spector et al., 2006) and the behavioral relevance of discriminating highly similar inputs (Schoups et al., 2001). In early readers, written words – but not visually unfamiliar false font strings – may already place sufficient demands on fine-grained perceptual discrimination to engage adaptation mechanisms reliably.

Although non-linguistic adaptation was largely preserved at the whole-brain level, our data suggested some evidence for a developmental divergence in face adaptation within the right FFA ROI. Namely, typically developing children showed increased face adaptation from kindergarten to third grade, whereas children with dyslexia did not, resulting in a significant group × time interaction. However, this interaction was observed only in group-defined FFA ROI (both matched and the entire longitudinal sample). It did not replicate in the individually-defined FFA ROI, though in the entire longitudinal sample a time effect was observed. Across the full sample, face adaptation in the group-defined, but not individually-defined, right FFA was negatively associated with phonological awareness. This pattern suggests that the developmental specialization of the high-level visual cortex may be differentially coupled to language-related skills. Within interactive specialization frameworks (Johnson, 2011), cortical regions compete and cooperate for representational resources as experience shapes functional networks. From this perspective, increased specialization for faces may be associated with weaker phonological representations, reflecting differences in how visual and language systems become calibrated during development.

Taken together, the present findings support a nuanced view of neural adaptation in dyslexia. Reduced adaptation to written words in the left vOT appears early, prior to formal reading acquisition, and relates dimensionally to pre-reading skills, consistent with its potential role as a contributing factor to reading difficulties. Moreover, a complementary individually-defined ROI approach revealed an anatomical specificity of this effect, which was confined to the anterior vOT. In contrast, there is little evidence for a domain-general adaptation deficit. Instead, developmental differences were restricted to specific visual categories and cortical regions, rather than evidence for a uniform impairment in neural adaptation.

Several limitations should be acknowledged. Despite careful case–control matching and preregistration, sample sizes were modest, particularly for longitudinal analyses, limiting power to detect small effects and interactions. Whole-brain null results should therefore be interpreted cautiously. We found no evidence for significant changes in the strength of neural adaptation to words with time and reading instruction. Since the study was conducted during the COVID-19 pandemic, only the scans performed in kindergarten and third grade had sufficient attendance for analysis. Therefore, we may have missed intermediate changes, as visual print specialization is thought to follow an inverted U-shaped trajectory – peaking early in reading instruction and declining with expertise (Price and Devlin, 2011, Maurer et al., 2006, Fraga-González et al., 2021, Chyl et al., 2021, Dehaene-Lambertz et al., 2018). It is therefore possible that our measurement points did not capture the peak of this adaptation curve. Future studies with larger samples and additional time points will be critical for modeling developmental trajectories and testing whether early neural adaptation predicts later reading outcomes beyond behavioral risk markers.

In conclusion, the present preregistered longitudinal study identifies small, stimulus-specific differences in neural adaptation to written words within left ventral occipito-temporal cortex prior to formal reading instruction. These effects were observed only in region-of-interest analyses and were not accompanied by widespread whole-brain differences, underscoring their subtle and localized nature. While modest associations with language-related skills suggest links between visual specialization and early literacy development, these relationships remain correlational and should be interpreted cautiously. Replication in larger longitudinal samples will be essential for determining the robustness and developmental significance of these effects. This work highlights the value of preregistered longitudinal designs for detecting subtle neural differences associated with later reading outcomes.

## CRediT authorship contribution statement

**Anna Banaszkiewicz:** Writing – review & editing, Writing – original draft, Methodology, Formal analysis, Conceptualization. **Katarzyna Chyl-Tanaś:** Writing – review & editing, Methodology, Investigation, Conceptualization. **Hanna Górecka:** Writing – review & editing, Visualization, Formal analysis. **Karolina Zielińska:** Writing – review & editing, Formal analysis. **Anna Redeł:** Writing – review & editing, Formal analysis. **Anna Szukało:** Writing – review & editing, Formal analysis. **Agnieszka Dębska:** Writing – review & editing, Writing – original draft, Investigation, Conceptualization. **Katarzyna Jednoróg:** Writing – review & editing, Writing – original draft, Supervision, Resources, Methodology, Funding acquisition, Formal analysis, Conceptualization.

## Ethics statement

The study was approved by the Research Ethics Committee at the SWPS University of Social Sciences and Humanities in Warsaw (date of approval 13/10/2015, approval number 28/2015). Parents of children who participated signed an informed consent form, and children agreed verbally. Families were reimbursed for participation in the project.

## Declaration of Competing Interest

The authors declare no competing interests.

## Data Availability

Behavioral, ROI, and second-level neuroimaging data and code are available at OSF: https://osf.io/ytfcv. Any additional information required to analyze the data reported in this paper is available on request.
