# Supplementary material for: Early differences in neural adaptation to written words before reading instruction in dyslexia
Source: Dev Cogn Neurosci. 2026 Sep 10;81:101815. doi: 10.1016/j.dcn.2026.101815 (PMC13594855; doi:10.1016/j.dcn.2026.101815)
Supplement: Supplementary file 1 [file mmc1.docx]

**Supplementary Materials**

1. **Pre-registration deviations**

Following deviations from the pre-registered protocol resulted from anonymous reviewers’ feedback during the initial peer-review process.

*1.1. Participants*

Reviewers raised concern about early between-group differences in reading scores at TP1, indicating that some children (primarily in the control group) were no longer pre-readers and they significantly differed in their reading performance from the dyslexic group (see section 3.1.1 below). As a result, it was impossible to determine whether adaptation deficits are a cause or a consequence of reading difficulties. Thus, we decided to use a case-control matching in SPSS to match the dyslexic and control groups in terms of age and word reading score at TP1. The procedure and results can be found in the *Participants* section in the main text. The results of the whole pre-registered sample are reported in Supplementary Materials 3 (below).

## *1.2. fMRI data preprocessing*

We addressed the reviewer's concern regarding lenient movement criteria. Namely, we revisited the decision to allow runs with up to 20% of volumes exceeding the ART Toolbox threshold and reduced this maximum to 10%. As suggested by the reviewer, we also now include additional information on movement (see sections 2.1.1 and 2.2.1 below).

*1.3. First-level (individual) analysis*

Following the comment from one of the reviewers that oddball trials and button press time-windows might contribute to unexplained variance and potential biases, we modelled timings of trials with red dots together with following interstimuli intervals (1200 ms for each target trial) as regressors of no interest.

*1.4. ROI analysis*

*1.4.1. Group-defined ROIs*

The pre-registered LEX ROI covered the posterior part of the ventral occipitotemporal cortex (vOT, see Figure S1). However, developmental models of reading propose that preschool and early elementary children develop their literacy skill from the letter-level alphabetic stage to the word-level orthographic stage (e.g., Frith, 1985). While the small-grain letter-to-phone mapping has been associated with the posterior vOT activity, the large-grain orthography-to-phonology mapping has been linked to activity in the anterior vOT (see Wang et al., 2021). To capture these developmental transitions, we replaced the LEX ROI with the vOT ROI encompassing both anterior and posterior portions of the vOT (Figure S1) by using a procedure analogous to that used for creating the FFA and LOC ROIs. Namely, we defined the left vOT ROI as a intersection of an independent left vOT mask from Dębska et al. (2026) and the overall activation mask of *Words_mix > baseline* from the total sample (at p<0.001 uncorrected; see Figure S1). Then, we created its right-hemispheric homologue using the MATLAB *flip* function.

Figure S1. Overlap of the pre-registered left LEX ROI (red) and the group-defined left vOT ROI (yellow).

*1.4.2. Individually-defined ROIs*

During the review process, we were asked by the anonymous reviewer to perform additional individually-defined ROI analyses, accounting for inter-individual variability in the location of category-selective cortex, which are now presented as a complementary approach.

First, we defined a common left and right vOT search space based on the group-level *Words_mix* > *baseline* contrast averaged across TP1 and TP2. This search space was intentionally broad, encompassing both the classical Visual Word Form Area (VWFA) and the more posterior word-responsive regions observed in children with dyslexia while excluding the cerebellum (using all cerebellum anatomical masks from AAL3 atlas combined in ImCalc) from the original mask. The search space thus served only to constrain the possible location of the individual ROIs rather than as the ROI itself.

The initial group-derived search space showed a bimodal posterior–anterior distribution, with a large posterior component centered in the occipital/lingual cortex and a second component located in posterior-to-mid ventral occipitotemporal cortex with similar distributions in the left and right vOT. Therefore, we decided to divide this search space to more anterior regions (y>-70) and more posterior regions (y<-70; see Figure S2).

Figure S2. A bimodal (posterior-anterior) distribution of voxels within the A) left vOT and B) right vOT.

In case of the left vOT the ROI sizes did not differ between the groups as tested with Welch t-test: anterior (Control Mean = 96.64, SD = 16.80, range = 16-100; Dyslexic Mean = 93.50, SD = 21.51, range = 14-100; t(39.61) = 0.552, p = 0.584); posterior (Control Mean = 93.04, SD = 22.36, range = 2-100; Dyslexic Mean = 85.82, SD = 29.96, range = 11-100; t(38.54) = 0.926, p = 0.36) for the total sample. Neither the ROI sizes differ for the matched samples: anterior (Control Mean = 94.75, SD = 21.5, range = 16-100; Dyslexic Mean = 94.63, SD = 21, range = 14-100; t(29.98) = 0.017, p = 0.987); posterior (Control Mean = 89.13, SD = 27.37, range = 2-100; Dyslexic Mean = 89.19, SD = 29.56, range = 11-100; t(29.84) = -0.006, p = 0.995). In case of the right vOT for the total sample the ROI sizes differed significantly for the anterior ROI with Controls having more word-selective voxels than Dyslexics (Control Mean = 86.52, SD = 29.54, range = 16-100; Dyslexic Mean = 53.86, SD = 45.91, range = 0-100; t(35.02) = 2.856, p = 0.007). There were no differences for the right posterior vOT (Control Mean = 73.64, SD = 38.35, range = 0-100; Dyslexic Mean = 74.50, SD = 35.31, range = 0-100; t(44.90) = -0.080, p = 0.937). For matched sample, the difference in the size of the anterior ROI was still present (Control Mean = 88.94, SD = 26.74, range = 16-100; Dyslexic Mean = 55.25, SD = 44.16, range = 0-100; t(24.69) = 2.61, p = 0.015), while there was no difference in the size of the posterior ROI (Control Mean = 68.19, SD = 41.36, range = 0-100; Dyslexic Mean = 75.25, SD = 33.12, range = 2-100; t(28.63) = -0.53, p = 0.598). Only in the left FFA 1 control subject had less than 100 voxels (87 voxels), for other ROIs (right FFA, left and right LOC) all subjects had 100 voxels selected. For the overlap of the individually-defined ROIs in the control and dyslexic groups see Figure S3.

Figure S3. Overlap of the individually-defined ROIs in the control (red) and dyslexic (green) groups. The scale represents the number of subjects with overlapping voxels.

*1.4.3. Non-preregistered exploratory ROI analysis*	One reviewer pointed out the lack of longitudinal ROI analysis for all ROIs. Thus, we performed exploratory two-way ANOVAs with a within-subject factor of TP and a between-subject factor of group for adaptation to words in the bilateral vOT, adaptation to faces in the bilateral FFA, and adaptation to objects in the bilateral LOC. Results for the matched sample are reported in the main text (*ROI results* section). Exploratory ROI analyses were also performed for the original sample (see Supplementary Materials 3.1.3).

*1.5. Second-level (group) analysis*

Pre-registered thresholds for reporting significant clusters were chosen based on Perrachione et al. (2016): a more lenient voxel-wise *p*<0.05 and cluster-wise *p*<0.05 false discovery rate corrected (FDRc) for between-group comparisons and a more stringent voxel-wise *p*<0.001, FDRc *p*<0.05 corrected for within-group comparisons. However, during data analysis we found out that the FDRc thresholding leads to losing meaningful information, which could be potentially detrimental for future replicability (see Taylor et al., 2023). Additionally, methodological work (e.g., Eklund et al., 2016) has demonstrated that more lenient voxel-wise thresholds such as *p*<0.05 can substantially inflate false positive rates in cluster-based inference. To align with current best practices, we decided to report all results using a more conservative voxel-wise threshold of *p*<0.001, combined with a cluster-wise p<0.05 family-wise error correction (FWEc). These results were complemented with the pre-registered FDRc correction, if FWE and FDR cluster extents differed.

To ensure that the observed effects reflected differences in activation, rather than deactivation (Frankenstein et al., 2003), group-level results were masked with a combined positive activation image. First-level contrasts (category_mix + category_adapt) at each TP were entered into one-sample t-tests across all participants (voxel-level p<0.05), binarized, and summed using ImCalc to create the final mask. The mask was applied only for visualization purposes and was not used during model estimation.

**2. Movement analysis and button press analysis**

2.1. Longitudinal analysis of 16 matched pairs (see Main text):

*2.1.1. Movement analysis:*

Both groups had on average 3.91 usable runs included in the analysis. A two-sample t-test showed no significant differences in the total number of censored volumes between groups at both TPs (TP1: *t*(30) = 0.65, *p* = 0.518, *M* dyslexic group = 4.25, *M* control group = 5.75; TP2: *t*(30) = -1.95, *p* = 0.059, *M* dyslexic group = 5.62, *M* control group = 1.88.

*2.1.2. Responses to target trials:*

Two participants (1 dyslexic and 1 control) were excluded from the analysis due to missing responses during at least two runs. One control participant was excluded because of a high rate of responses in each of the runs (between 53 and 178, where the stimulus was presented only 10 times per run). The absence of responses or an unusually high number of button presses might suggest a lack of understanding or adherence to task instructions or technical issues with the response pad.
Mann-Whitney U tests were performed due to not normal distribution. Results showed no differences in the number of correct responses to target trials between groups at TP1(*W* = 87.5, *p* = 0.456, *M* dyslexic group = 86%, *M* control group = 83%) and at TP2 (*W* = 121, *p* = 0.457, *M* dyslexic group = 96%, *M* control group = 98%). Groups did not differ significantly in the reaction times (TP1: *W* = 128, *p* = 0.331, *M* dyslexic group = 0.762 ms, *M* control group = 0.801 ms; TP2: *W* = 109, *p* = 0.881, *M* dyslexic group = 0.607 ms, *M* control group = 0.615 ms).

2.2. Longitudinal analysis of the entire longitudinal sample (see Supplementary Material below):

*2.2.1. Movement analysis:*

The control group had on average 3.92 runs included in the analysis, while participants in the dyslexia group had *M* = 3.89 usable runs. A two-sample t-test showed no significant difference in the total number of censored volumes between groups at both TPs (TP1: *t*(45) = -0.27, *p* = 0.791, *M* dyslexic group = 6.73, *M* control group = 6.0; TP2: *t*(45) = -1.30, *p* = .0.199, *M* dyslexic group = 5.55, *M* control group = 3.16).

*2.2.2. Responses to target trials:*

Four participants (1 dyslexic and 3 control) were excluded from the analysis due to missing responses during at least two runs. Two control participants were excluded because of a high rate of responses in each of the runs.
Mann-Whitney U tests showed no differences in the number of correct responses to target trials between groups at TP1(*W* = 216.5, *p* = 0.875, *M* dyslexic group = 87%, *M* control group = 85%) and at TP2 (*W* = 247.5, *p* = 0.284, *M* dyslexic group = 95%, *M* control group = 99%). Groups did not differ significantly in the reaction times (TP1: *W* = 254, *p* = 0.260; *M* dyslexic group = 0.751 ms, *M* control group = 0.786 ms; TP2: *W* = 197, *p* = 0.747; *M* dyslexic group = 0.600 ms, *M* control group = 0.604 ms).

**3. Results for the entire longitudinal sample**

3.1. Below we provide results for the entire longitudinal sample of dyslexic (N = 22) and control (N = 25) participants at TP1 and TP2.

*3.1.1. Behavioral longitudinal results*

For word reading, there were significant main effects of group (*F*(1,44) = 74.45, *p*<0.001, η_p_^2^ = 0.63), TP (*F*(1,44) = 409.30, *p*<0.001, η_p_^2^ = 0.90), and interaction of group x TP (*F*(1,44) = 46.57, *p*<0.001, η_p_^2^ = 0.51). For pseudoword reading analysis showed analogous results: a main effect of the group (*F*(1,44) = 36.49, *p*<0.001, η_p_^2^ = 0.45), TP (*F*(1,44) = 312.26, *p*<0.001, η_p_^2^ = 0.88), and interaction of group x TP (*F*(1,44) = 10.15, *p*=0.003, η_p_^2^ = 0.19). The post-hoc analysis revealed that control children read significantly more words than dyslexic children at both TPs (TP1: *t*(44) = 3.95, *p* = 0.002, *M* dyslexic group = 3.1, *M* control group = 11.2; TP2: *t*(44) = 8.28, *p*<0.001, *M* dyslexic group = 40.2, *M* control group = 86.0). Moreover, both groups showed a significant improvement in word reading from TP1 to TP2 (control group: *t*(44) = 20.02, *p*<0.001; dyslexic group: *t*(44) = 9.09, *p*<0.001), though it was more pronounced in control children. Similarly, for the pseudoword reading, control children performed significantly better compared to dyslexic children at both TPs (TP1: *t*(44) = 3.60, *p*=0.005, *M* dyslexic group = 2.8, *M* control group = 9.8; TP2: *t*(44) = 5.41, *p*<0.001, *M* dyslexic group = 29.4, *M* control group = 48.2). Both groups improved significantly in pseudoword reading from TP1 to TP2 (control group: *t*(44) = 15.44, *p*<0.001; dyslexic group: *t*(44) = 9.82, *p*<0.001), with more pronounced changes in the controls. See Figure S4 for details.

The groups did not differ significantly in IQ, or age at TP2, but a small difference in age was present at TP1 (with the control group being older than the dyslexic group). Control children outperformed children with dyslexia at TP1 on letter knowledge, PA and RAN, while no group differences were found for visual attention. See Table S1 for details.

Figure S4. Word and pseudoword reading performance in the control and dyslexic group at two time points (TP). Black dots indicate mean values. Significant differences between groups and TPs are indicated with asterisks, *** *p*<0.001, ** *p*<0.01.

Table S1. Demographics at TP1 and TP2 and test performance at TP1 (kindergarten) in the control and dyslexic group.

|  | Control  n=25 | | | Dyslexic  n=22 | | | Independent samples test | |
| --- | --- | --- | --- | --- | --- | --- | --- | --- |
|  | Mean | SD | Range | Mean | SD | Range | t | p |
| Age T1 | 6.83 | 0.34 | 6.25 - 7.38 | 6.59 | 0.47 | 5.50 - 7.31 | 2.07 | 0.044 |
| Age T2 | 9.88 | 0.29 | 9.22 - 10.48 | 9.71 | 0.43 | 8.83 - 10.48 | 1.57 | 0.123 |
| Cattel IQ | 109.42 | 13.15 | 77 - 132 | 88 | 14.34 | 88 - 147 | -0.55 | 0.587 |
| Phonological awareness PC | 0.43 | 0.94 | -1.72 - 1.87 | -0.39 | 0.94 | -1.72 - 1.64 | 2.96 | 0.005 |
| RAN (sec) | 79.10 | 21.01 | 52-140 | 92.41 | 1938 | 63 - 128.5 | -2.25 | 0.03 |
| Letter knowledge (upper & lower case) | 49.52 | 11.55 | 20 - 64 | 33.0 | 16.02 | 4 - 63 | 4.09 | <0.001 |
| Visual Attention  (max score 105) | 37.42 | 10.84 | 17 - 62 | 37.27 | 10.10 | 15 - 54 | 0.05 | 0.963 |

*3.1.2. Whole-brain fMRI longitudinal results*

In controls, word adaptation appeared at TP1 in the bilateral ventral occipito-temporal cortex, including fusiform gyrus. At TP2 adaptation in the left ventral occipito-temporal remained, whereas in the right hemisphere it was reduced to the occipital cortex. In dyslexia, word adaptation was seen only at TP2 in bilateral inferior and middle occipital cortex. Neither group showed adaptation to false fonts. Face adaptation was found at TP2 in controls (bilateral fusiform, occipital, right temporal gyri, left cerebellum) and at both TPs in dyslexia (bilateral occipito-temporal regions and left lingual gyrus at TP1, restricted to the right hemisphere at TP2). House adaptation was present at TP1 in dyslexia (right fusiform and parahippocampal gyrus) and at TP2 in controls (bilateral fusiform, occipital, right lingual gyrus, left cerebellum). Object adaptation occurred in controls (bilateral fusiform, inferior occipito-temporal gyri, left cerebellum) and dyslexia (bilateral fusiform, occipito-temporal, right parahippocampal) at both TPs (see Figure S5, Table S2 for details).

Figure S5. Adaptation to visual stimuli categories in control children and children with dyslexia at TP1 and TP2. All contrasts at voxelwise *p <* 0.001 and clusterwise *p <* 0.05 FWE corrected.

Table S2. Neurophysiological adaptation to different categories of stimuli in control and dyslexic children at TP1 (kindergarten) and TP2 (3rd grade). Only significant effects at voxelwise p<0.001, and clusterwise *p <* 0.05 FWE corrected.

|  | |  |  | MNI Coordinates | | |
| --- | --- | --- | --- | --- | --- | --- |
| Contrast Name | Region Label | Extent | t-value | x | y | z |
| **TP1** | | | | | | |
| Control group: words | Occipital_Inf_R, Fusiform_R, Temporal_Inf_R, Cerebelum_6_R | 409 | 4.85 | 36 | -50 | -22 |
|  | Fusiform_L, Occipital_Inf_L, Temporal_Inf_L, Cerebelum_Crus1_R | 718 | 4.69 | -36 | -52 | -16 |
| Control group: objects^a^ | Fusiform_R, Occipital_Inf_R, Temporal_Inf_R, Occipital_Mid_R | 2673 | 9.55 | 34 | -40 | -22 |
|  | Fusiform_L, Occipital_Inf_L, Occipital_Mid_L, Temporal_Inf_L, Cerebelum_6_L | 2566 | 9.80 | -30 | -56 | -18 |
| Dyslexic group: faces | Fusiform_R, Temporal_Inf_R, | 460 | 6.79 | 42 | -46 | -22 |
|  | Occipital_Inf_R, Fusiform_R, Temporal_Inf_R, Temporal_Mid_R | 346 | 5.06 | 44 | -74 | -6 |
|  | Fusiform_L, Temporal_Inf_L | 387 | 7.96 | -36 | -48 | -18 |
|  | Occipital_Inf_L, Fusiform_L, Lingual_L, Occipital_Mid_L | 239 | 5.29 | -32 | -72 | -12 |
|  |  |  |  |  |  |  |
| Dyslexic group: houses^b^ | Fusiform_R, ParaHippocampal_R | 504 | 7.54 | 36 | -52 | -6 |
| Dyslexic group: objects^c^ | Fusiform R, Occipital_Inf_R, Occipital_Mid_R, Temporal_Inf_R, ParaHippocampal_R | 3366 | 9.23 | 32 | -38 | -14 |
|  | Fusiform L, Occipital_Mid_L, Occipital_Inf_L | 2732 | 8.76 | -38 | -78 | -10 |
| **TP2** | | | | | | |
| Control group: words^d^ | Occipital_Inf_R, Occipital_Mid_R | 327 | 4.74 | -38 | -84 | -4 |
|  | Occipital_Inf_L, Fusiform_L, Occipital_Mid_L, Temporal_Inf_L | 1495 | 6.41 | 38 | -86 | -4 |
| Control group: faces^e^ | Fusiform_R, Occipital_Inf_R, Occipital_Mid_R, Temporal_Inf_R | 1561 | 6.91 | 38 | -42 | -16 |
|  | Fusiform_L, Occipital_Inf_L, Occipital_Mid_L, Cerebelum_6_L | 1241 | 5.40 | -36 | -46 | -24 |
|  |  |  |  |  |  |  |
| Control group: houses | Fusiform_R, Occipital_Mid_R, Lingual_R, Occipital_Inf_R | 1878 | 9.52 | 32 | -58 | -8 |
|  | Fusiform_L, Occipital_Inf_L, Cerebelum_6_L | 665 | 7.87 | -30 | -52 | -16 |
|  |  |  |  |  |  |  |
| Control group: objects^f^ | Fusiform_R, Occipital_Mid_R, Occipital_Inf_R, Temporal_Inf_R | 4342 | 9.57 | 32 | -92 | 10 |
|  | Occipital_Mid_L, Fusiform_L, Occipital_Inf_L, Cerebelum_6_L | 3587 | 9.32 | -34 | -44 | -18 |
|  |  |  |  |  |  |  |
| Dyslexic group: words^g^ | Occipital_Inf_R, Occipital_Mid_R, Temporal_Inf_R | 842 | 7.91 | 42 | -84 | -10 |
|  | Occipital_Inf_L, Occipital_Mid_L | 747 | 6.09 | -34 | -84 | -10 |
|  |  |  |  |  |  |  |
| Dyslexic group: faces^h^ | Fusiform_R, Occipital_Inf_R, Temporal_Inf_R | 270 | 5.72 | 38 | -44 | -16 |
| Dyslexic group: objects^i^ | Occipital_Inf_R, Temporal_Inf_R | 362 | 5.72 | 36 | -66 | -6 |
|  | Fusiform_L, Temporal_Inf_L | 503 | 5.65 | -32 | -44 | -18 |
|  | Occipital_Mid_L, Occipital_Inf_L | 373 | 5.03 | 30 | -98 | 4 |
| Note: Regions were automatically labeled using the AAL3 with a minimum contribution of 5% to the clusters, x, y, and z =Montreal Neurological Institute (MNI) coordinates in the left-right, anterior-posterior, and inferior-superior dimensions, respectively. ^a^FDR cluster extent=not significant, ^b^FDR cluster extent=not significant, ^c^FDR cluster extent=3366, ^d^FDR cluster extent=1495, ^e^FDR cluster extent=1561, ^f^FDR cluster extent=4342, ^g^FDR cluster extent=842, ^h^FDR cluster extent=not significant, ^i^FDR cluster extent=373 | | | | | | |

No significant main effect of time point or group × TP interaction was found. A two-sample t-test examining the main effect of group on neural adaptation to words revealed a cluster in the left fusiform gyrus, with control children showing more adaptation than dyslexic group. Given our a priori hypothesis about reduced word-specific adaptation in the left ventral occipito-temporal cortex in dyslexia, we applied a small volume correction (SVC) using the left vOT ROI. We observed a cluster of 125 voxels, *t* = 4.08, *p*=0.002 FWEc, *p*=0.026 FDRc; see Figure S6). No other main effects of group were found.

Figure S6. Main effect of group in the left vOT ROI in whole brain analysis with children with dyslexia showing lower adaptation to words than typical readers.

**3.1.3. Exploratory ROI results for the entire longitudinal sample**

*Group-defined ROIs*
Additionally, we performed exploratory mixed-design ANOVAs with a within-subject factor of TP and a between-subject factor of group for adaptation to words in the bilateral vOT, adaptation to faces in the bilateral FFA, and adaptation to objects in the bilateral LOC. Results revealed significant effects only in the case of adaptation to words in the left vOT: a significant effect of group (*F*(1,45) = 13.44, *p*<0.001, η_p_^2^ = 0.230) was revealed with stronger adaptation to words in the control than the dyslexic group, while the effect of time (*F*(1,45) = 0.08, *p*=0.781) and time x group interaction (*F*(1,45) = 0.38, *p*=0.539) were not significant. In the right FFA, only a group x time interaction (*F*(1,45) = 6.48, *p*=0.014, η_p_^2^ = 0.13) was observed, but not main effects of group (*F*(1,45) = 1.06, *p*=0.309) and time (*F*(1,45) = 2.51, *p*=0.120). Post-hoc analysis revealed that only the control group presented a significant increase of the neural adaptation with time (*t*(45) = 3.02, p=0.025), while it was not present in the dyslexic group (*t*(45) = 0.66, *p*=1.00). See Figure S7.

Figure S7. Results for group-defined ROI analyses: adaptation to words in the bilateral vOT, adaptation to faces in the bilateral FFA, and adaptation to objects in the bilateral LOC. A significant main effect of the group in the left vOT and a significant difference between TPs within the control group in the right FFA are indicated with asterisks, * *p<*0.05, *** *p<*0.001.

*Individually-defined ROIs*

Results revealed a significant effect in the case of adaptation to words in the left anterior vOT and adaptation to faces in the right FFA. For the left anterior vOT, a significant main effect of group (*F*(1, 45) = 9.08, *p* = .004, ηp² = .168) was found with stronger adaptation to words in the control than the dyslexic group. There was no significant main effect of time point, *F*(1, 45) = 0.02, *p* = .901, ηp² < .001, nor the group × time point interaction, *F*(1, 45) = 0.53, *p* = .469, ηp² = .012. In the right FFA, there was a significant main effect of time point (*F*(1, 45) = 4.14, *p* = .048, ηp² = .084), indicating an overall increase in face adaptation from TP1 to TP2 across both groups. There was no no significant main effect of group (*F*(1, 45) = 2.28, *p* = .138, ηp² = .048), and no group × time point interaction (*F*(1, 45) = 1.51, *p* = .225, ηp² = .033). No significant effects were observed in the left posterior vOT, in the right anterior or posterior vOT, left FFA and LOC (see Figure S8).

Figure S8. Results for individually-defined ROI analyses: adaptation to words in the bilateral vOT, adaptation to faces in the bilateral FFA, and adaptation to objects in the bilateral LOC. A significant main effect of the group in the left vOT and a significant time effect in the right FFA are indicated with asterisks, * *p<*0.05, ** *p<*0.01.

**4. Results for the cross-sectional sample**

4.1. Below we provide results for the entire cross-sectional sample (both groups pooled together, N = 59) at TP1.

*4.1.1. Behavioral results*

For demographics and test performance in the entire sample of children tested at TP1 see Table S3.

Table S3. Demographic characteristics and test performance during TP1 (kindergarten) in the entire sample of 59 children.

|  | Mean | SD | Range |
| --- | --- | --- | --- |
| Age TP1 | 6.73 | 0.39 | 5.5 - 7.38 |
| Cattel IQ | 110.22 | 13.64 | 77 - 147 |
| Phonological awareness PC | 0.03 | 0.96 | -1.72 - 1.87 |
| RAN (sec) | 83.37 | 19.79 | 52-140 |
| Letter knowledge (upper & lower case) | 39.62 | 17.27 | 4 - 64 |
| Visual Attention (max score 105) | 37.83 | 9.68 | 15 - 62 |
| Word Reading (per min) | 6.68 | 7.48 | 0 - 35 |
| Pseudoword Reading (per min) | 6.08 | 7.08 | 0 - 32 |

We performed correlations between pre-reading skills and demographic variables (age, IQ). Age was weakly positively related to word and pseudoword reading and negatively with IQ. IQ showed weak positive correlation with PA and moderate positive correlation with visual attention. PA was strongly positively correlated with letter knowledge, word and pseudoword reading. RAN showed weak negative correlation with visual attention only. Letter knowledge correlated strongly with word and pseudoword reading (for details see Table S4).

Table S4. Pearson’s Correlations between age, IQ and behavioral scores in the entire sample of 59 children.

| Variable | 1. | 2. | 3. | 4. | 5. | 6. | 7. |
| --- | --- | --- | --- | --- | --- | --- | --- |
| 1. Age^#^ | – |  |  |  |  |  |  |
| 2. IQ | -0.36^*^ | – |  |  |  |  |  |
| 3. Phonology_PC | 0.07 | 0.28^*^ | – |  |  |  |  |
| 4. RAN_avg | -0.14 | -0.25 | -0.23 | – |  |  |  |
| 5. Visual attention | 0.04 | 0.37^**^ | 0.16 | -0.30^*^ | – |  |  |
| 6. Letters | 0.19 | 0.09 | 0.55^***^ | -0.08 | 0.05 | – |  |
| 7. Word Reading | 0.32^*^ | 0.14 | 0.64^***^ | -0.19 | 0.25 | 0.69^***^ | – |
| 8. Pseudoword Reading | 0.28^*^ | 0.13 | 0.65^***^ | -0.20 | 0.19 | 0.72^***^ | 0.94^***^ |

Note: ^#^Age_Screening for RAN_avg; Age_Testing for Phonology_PC, Visual attention, Letters, IQ; Age_MRI_T1 for Word reading; ^*^p<0.05, ^**^p<0.01, ^***^p<0.001 (two-tailed uncorrected)

*4.1.2. Whole-brain and ROI x behavioral results*

A cross-sectional fMRI-RA analysis showed adaptation to words in bilateral fusiform and occipital cortex as well as the right temporal inferior gyrus. Adaptation to the false font and faces was found in bilateral fusiform, occipital and temporal regions and the right lingual gyrus. In addition to bilateral fusiform and occipital gyri house adaptation was found also in parahippocampal gyri. Object adaptation was observed bilaterally in the fusiform, occipital and temporal gyri, as well as in the left cerebellum. See Figure S9A and Table S5.

Figure S9. A) Adaptation to all visual categories in the entire sample (N = 59) at TP1. All contrasts are shown at voxel-wise p < 0.001 and cluster-wise p < 0.05, FWE-corrected. B) Neural adaptation to words in the group-defined left vOT was positively associated with word (and pseudoword) reading as well as with phonological awareness. C) Neural adaptation to words in the individually-defined left anterior vOT was positively associated with word reading.

Table S5. Neurophysiological adaptation to different categories of stimuli in the entire sample (control and dyslexic children, N = 59) at Time Point 1 (kindergarten). Only significant effects at voxelwise *p*<0.001, and clusterwise *p<*0.05 FWE corrected.

|  | |  |  | MNI Coordinates | | |
| --- | --- | --- | --- | --- | --- | --- |
| Contrast Name | Region Label | Extent | t-value | x | y | z |
| **TP1 (entire sample)** | | | | | | |
| Words | Occipital_Inf_R, Fusiform_R, Temporal_Inf_R, Occipital_Mid_R | 1444 | 5.49 | 36 | -84 | -4 |
|  | Occipital_Inf_L, Fusiform_L | 369 | 4.73 | -34 | -50 | -18 |
| False font^a^ | Fusiform_R, Occipital_Inf_R, Temporal_Inf_R, Lingual_R | 1201 | 5.40 | 36 | -74 | -10 |
|  | Occipital_Inf_L, Fusiform_L, Temporal_Inf_L | 508 | 4.51 | -40 | -70 | -12 |
| Faces | Fusiform_R, Occipital_Inf_R, Occipital_Mid_R, Temporal_Inf_R, Lingual_R | 1847 | 6.61 | 44 | -76 | -6 |
|  | Fusiform_L, Occipital_Inf_L, Occipital_Mid_L | 1218 | 6.92 | -34 | -48 | -18 |
| Houses^b^ | Fusiform_R, Occipital_Inf_R, ParaHippocampal_R, Temporal_Inf_R | 1313 | 6.90 | 32 | -40 | -8 |
|  | Fusiform_L, Occipital_Inf_L, ParaHippocampal_L | 770 | 6.22 | -32 | -60 | -8 |
|  |  |  |  |  |  |  |
| Objects^c^ | Fusiform_R, Occipital_Inf_R, Occipital_Mid_R, Temporal_Inf_R, | 5274 | 14.33 | 32 | -38 | -18 |
|  | Fusiform_L, Occipital_Mid_L, Occipital_Inf_L, Temporal_Inf_L, Cerebelum_6_L | 5084 | 13.38 | -30 | -50 | -14 |
| Note: Regions were automatically labeled using the AAL3 with a minimum contribution of 5% to the clusters, x, y, and z =Montreal Neurological Institute (MNI) coordinates in the left-right, anterior-posterior, and inferior-superior dimensions, respectively. ^a^FDR cluster extent=1201, ^b^FDR cluster extent=1313, ^c^FDR cluster extent=not significant. | | | | | | |

*Cross-sectional analysis: brain-behavior correlations at TP1* *for each group separately*

In line with preregistration, we performed correlations between pre-reading skills and neural adaptation in all ROIs in control and dyslexic groups separately (see Tables S6-S9). Partially in line with our hypothesis, results showed significant associations between the left vOT and PA in the control group (for group- and individually-defined ROIs) and between the left vOT and reading skills in the dyslexic group (only for group-defined ROIs). A hypothesized association between ROIs and RAN was found for the right anterior vOT in the dyslexic group, but in the direction opposite to our hypothesis: higher activation was associated with longer RAN naming times (i.e., poorer performance). Contrary to our hypothesis these associations were not present between ROIs and letter knowledge. We also hypothesized a lack of significant relationship between ROIs and visual attention. This was largely supported with the exception of the significant correlation between the individually-defined right anterior and posterior vOT and visual attention skill in the control group.

Table S6. Spearman’s correlations between pre-reading skills and group-defined ROIs in the control group (n=32). PA = phonological awareness, RAN = rapid automatized naming.

| Variable | PA | RAN | Visual attention | Letters | Word Reading | Pseudoword Reading |
| --- | --- | --- | --- | --- | --- | --- |
| Words_L_VOT | 0.42* | -0.12 | 0.12 | 0.21 | 0.20 | 0.18 |
| Words_R_VOT | 0.39* | 0.04 | 0.04 | 0.02 | 0.06 | 0.09 |
| Faces_L_FFA | -0.09 | 0.07 | -0.02 | -0.19 | -0.31 | -0.14 |
| Faces_R_FFA | -0.16 | 0.02 | -0.08 | -0.08 | -0.22 | -0.02 |
| Objects_L_LOC | -0.14 | -0.09 | 0.15 | -0.13 | -0.14 | -0.16 |
| Objects_R_LOC | -0.32 | -0.03 | -0.07 | -0.10 | -0.13 | -0.13 |

Note: ^*^p<0.05 (two-tailed uncorrected).

Table S7. Spearman’s correlations between pre-reading skills and group-defined ROIs in children with dyslexia (n=27).

| Variable | PA | RAN | Visual attention | Letters | Word Reading | Pseudoword Reading |
| --- | --- | --- | --- | --- | --- | --- |
| Words_L_VOT | 0.13 | 0.05 | 0.26 | 0.15 | 0.40* | 0.40* |
| Words_R_VOT | 0.12 | 0.06 | 0.08 | 0.31 | 0.43* | 0.35 |
| Faces_L_FFA | -0.21 | 0.08 | -0.17 | -0.00 | -0.10 | -0.23 |
| Faces_R_FFA | -0.36 | 0.10 | -0.20 | 0.111 | 0.23 | 0.06 |
| Objects_L_LOC | -0.11 | -0.04 | -0.12 | -0.07 | -0.07 | -0.12 |
| Objects_R_LOC | 0.01 | -0.21 | -0.04 | 0.04 | 0.09 | -0.01 |

Note: ^*^p<0.05 (two-tailed uncorrected).

Table S8. Spearman’s correlations between pre-reading skills and individually-defined ROIs in the control group (n=32). PA = phonological awareness, RAN = rapid automatized naming.

| Variable | PA | RAN | Visual attention | Letters | Word Reading | Pseudoword Reading |
| --- | --- | --- | --- | --- | --- | --- |
| Words_L_aVOT | 0.41* | -0.13 | 0.12 | 0.14 | 0.18 | 0.15 |
| Words_L_pVOT | 0.38* | -0.10 | 0.31 | 0.07 | -0.01 | -0.03 |
| Words_R_aVOT | 0.04 | -0.25 | 0.45* | -0.02 | 0.15 | 0.09 |
| Words_R_pVOT | 0.10 | -0.31 | 0.53** | 0.16 | 0.04 | -0.03 |
| Faces_L_FFA | 0.07 | 0.24 | -0.14 | -0.08 | -0.25 | -0.07 |
| Faces_R_FFA | -0.15 | -0.03 | -0.13 | -0.21 | -0.30 | -0.11 |
| Objects_L_LOC | 0.11 | 0.15 | -0.12 | -0.24 | 0.12 | 0.03 |
| Objects_R_LOC | 0.29 | 0.26 | -0.00 | 0.04 | 0.15 | 0.10 |

Note: ^*^p<0.05 (two-tailed uncorrected), ^**^p<0.01 (two-tailed uncorrected).

Table S9. Spearman’s correlations between pre-reading skills and individually-defined ROIs in the dyslexic group (n=27). PA = phonological awareness, RAN = rapid automatized naming.

| Variable | PA | RAN | Visual attention | Letters | Word Reading | Pseudoword Reading |
| --- | --- | --- | --- | --- | --- | --- |
| Words_L_aVOT | -0.15 | 0.11 | 0.14 | 0.01 | 0.25 | 0.24 |
| Words_L_pVOT | -0.1 | 0.23 | 0.16 | 0.03 | 0.02 | 0.03 |
| Words_R_aVOT | 0.10 | 0.52* | -0.20 | 0.11 | 0.14 | 0.16 |
| Words_R_pVOT | 0.23 | 0.06 | -0.32 | 0.05 | -0.06 | -0.08 |
| Faces_L_FFA | -0.25 | 0.05 | -0.19 | 0.17 | -0.05 | -0.16 |
| Faces_R_FFA | -0.31 | 0.17 | -0.23 | 0.05 | 0.18 | 0.06 |
| Objects_L_LOC | 0.21 | -0.26 | 0.30 | -0.19 | -0.13 | 0.03 |
| Objects_R_LOC | 0.24 | -0.20 | 0.31 | -0.20 | -0.27 | -0.08 |

Note: ^*^p<0.05 (two-tailed uncorrected).

**References**

Dębska, A., Wang, S., Jednoróg, K., & Pattamadilok, C. (2026). Reading acquisition drives linguistic cross-modal convergence in the left ventral occipitotemporal cortex. *NeuroImage*, *326*, 121678. https://doi.org/10.1016/j.neuroimage.2025.121678

Eklund, A., Nichols, T. E., & Knutsson, H. (2016). Cluster failure: Why fMRI inferences for spatial extent have inflated false-positive rates. *Proceedings of the National Academy of Sciences of the United States of America*, *113*(28), 7900–7905. https://doi.org/10.1073/pnas.1602413113

Frankenstein, U., Wennerberg, A., Richter, W., Bernstein, C., Morden, D., Rémy, F., & Mcintyre, M. (2003). Activation and deactivation in blood oxygenation level dependent functional magnetic resonance imaging. *Concepts in Magnetic Resonance Part A: An Educational Journal*, *16*(1), 63-70.

Frith, U. (1985). Beneath the surface of developmental dyslexia. Are comparisons between developmental and acquiried disorders meaningful. *Surface Dyslexia. Londres: LEA*.

Taylor, P. A., Reynolds, R. C., Calhoun, V., Gonzalez-Castillo, J., Handwerker, D. A., Bandettini, P. A., Mejia, A. F. & Chen, G. (2023). Highlight results, don't hide them: Enhance interpretation, reduce biases and improve reproducibility. *Neuroimage*, *274*, 120138.

Wang, J., Joanisse, M. F., & Booth, J. R. (2021). Letter fluency in 7-8-year-old children is related to the anterior, but not posterior, ventral occipito-temporal cortex during an auditory phonological task. *Developmental Cognitive Neuroscience*, *47*, 100898.
